# Extracellular Vesicles in Organ Fibrosis: Mechanisms, Therapies, and Diagnostics

**DOI:** 10.3390/cells10071596

**Published:** 2021-06-25

**Authors:** David R. Brigstock

**Affiliations:** 1 Center for Clinical and Translational Research, The Research Institute at Nationwide Children’s Hospital, Columbus, OH 43205, USA; David.Brigstock@NationwideChildrens.org; 2 Department of Surgery, Division of Pediatric Surgery, The Ohio State University Wexner Medical Center, Columbus, OH 43212, USA

**Keywords:** fibrosis, fibrogenic, extracellular vesicle, exosome, myofibroblast, collagen, extracellular matrix

## Abstract

Fibrosis is the unrelenting deposition of excessively large amounts of insoluble interstitial collagen due to profound matrigenic activities of wound-associated myofibroblasts during chronic injury in diverse tissues and organs. It is a highly debilitating pathology that affects millions of people globally and leads to decreased function of vital organs and increased risk of cancer and end-stage organ disease. Extracellular vesicles (EVs) produced within the chronic wound environment have emerged as important vehicles for conveying pro-fibrotic signals between many of the cell types involved in driving the fibrotic response. On the other hand, EVs from sources such as stem cells, uninjured parenchymal cells, and circulation have in vitro and in vivo anti-fibrotic activities that have provided novel and much-needed therapeutic options. Finally, EVs in body fluids of fibrotic individuals contain cargo components that may have utility as fibrosis biomarkers, which could circumvent current obstacles to fibrosis measurement in the clinic, allowing fibrosis stage, progression, or regression to be determined in a manner that is accurate, safe, minimally-invasive, and conducive to repetitive testing. This review highlights the rapid and recent progress in our understanding of EV-mediated fibrotic pathogenesis, anti-fibrotic therapy, and fibrosis staging in the lung, kidney, heart, liver, pancreas, and skin.

## 1. Introduction

Fibrosis is the production and deposition of excessively large amounts of collagenous scar material in the interstitial spaces. This process, which can occur in virtually every tissue or organ of the body, usually develops over a protracted time period (frequently measured in years) and is most often a response to chronic injury and is thus manifest as a highly exaggerated wound healing response. Pathways of fibrosis involve multiple cell types which communicate in an orchestrated fashion via intercellular signaling networks that involve numerous cell-associated, matricellular, or soluble signaling molecules. In recent years, a new mode of cell-cell communication has been identified that involves the delivery of molecular information in extracellular vesicles (EVs) and this is now considered to be a significant signaling mechanism by which many homeostatic and pathological processes are regulated. Emerging evidence shows that specific populations of EVs are stimulatory for fibrosis or may possess intrinsic or engineered anti-fibrotic properties; these aspects of EV biology are the subject of this review.

## 2. Fibrosis

**Figure 1:**
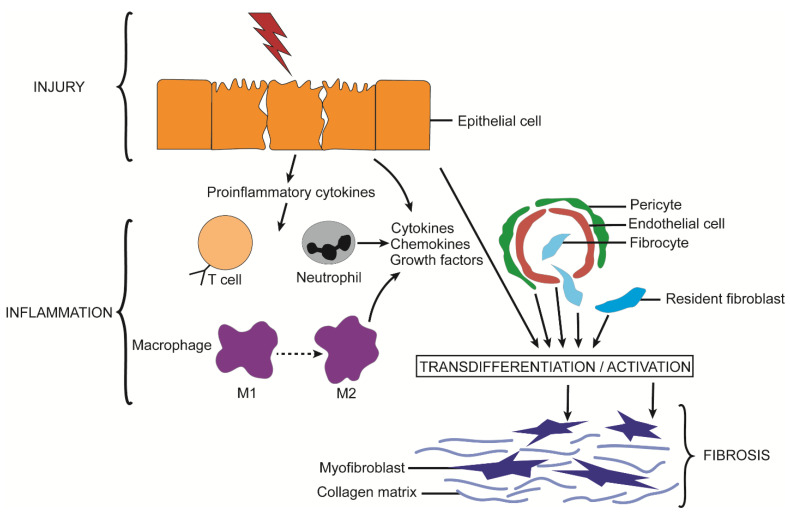
Principal cellular events leading to fibrosis. Injury to epithelial cells results in their release of pro-inflammatory cytokines, which is a stimulus for infiltration of macrophages and immune cells to the injury site. This results in an inflammatory environment in which cytokines, chemokines, and growth factors are produced that drive the production and action of contractile αSMA-producing myofibroblasts. Myofibroblasts arise by activation of resident fibroblasts or circulating fibrocytes or are the result of transdifferentiation from other cell types such as epithelial cells, endothelial cells, or pericytes. In acute injury, myofibroblasts transiently produce ECM components such as collagen, laminin, and FN which are necessary for normal wound healing, and parenchymal repopulation. During persistent or recurrent episodic injury, the inflammatory phase is protracted leading to unrelenting myofibroblastic activity, which is manifested as excessive production of ECM components that are deposited in the interstitial space as scar material, or fibrosis.

Fibrosis is a common feature of many varied chronic organ diseases in which it causes impairment of cell-cell communication, aberrant tissue remodeling, alterations in blood flow, reduced tissue or organ function, and increases the probability that more severe conditions such as end-stage organ disease or cancer will develop [1,2,3]. Fibrosis is the cause of considerable morbidity and mortality and has been estimated to contribute to 45% of all deaths in the USA [2,3]. While the etiology of fibrosis is multi-factorial and varies widely by organ, the molecular mechanisms that drive fibrosis often have many aspects in common [1,2,3]. The first event in the process is a typical wound healing response in which cell damage or death triggers a local inflammatory response, activation of tissue macrophages, and infiltration of immune cells from the circulation (Figure 1) [4]. In turn, cytokines and chemokines released by these cells are cues for the production of extracellular matrix (ECM) molecules by mesenchymal cells or myofibroblastic cells, the latter of which may be ordinarily resident in the tissue or arise by phenotypic transition from other cell types such as fibroblasts, epithelial cells, or endothelial cells [3]. This transition involves a process termed activation in which the cells become contractile, proliferative, migratory, and matrigenic and characteristically express high levels of alpha smooth muscle actin (αSMA), growth factors, integrins, chemokines, cytokines, and ECM components such as collagens (Figure 1). In acute injury, activation is relatively short-lived and the myofibroblastic cell population wanes as a provisional ECM is established for parenchymal repopulation and growth. In contrast, in chronic injury the population of activated cells persists unabated, resulting in an unrelenting production of insoluble collagens and other ECM molecules that become deposited in the intercellular spaces at high concentrations and over time are manifested as fibrotic scar (Figure 1) [1,2,3]. Many pro-fibrotic changes are driven by similar molecular pathways including activation of transforming growth factor beta (TGF-β) (and its small mothers against decapentaplegic (Smad) regulatory axis) [5,6,7,8,9,10,11,12,13,14], reactivation of developmental pathways such as wingless/integrated (Wnt), Notch, and Hedgehog (Hh) [15,16,17,18,19,20,21,22], and production of a similar slate of chemokines and growth factors [23]. Collectively, these signaling events conspire to drive fibroblast/myofibroblast differentiation and production of ECM molecules. As fibrosis progresses, it impedes normal cellular functions and may accelerate the underlying disease by exacerbating other pathological responses in the affected organ. Emerging evidence from in vitro studies, animal models, and some clinical studies have revealed that fibrosis is amenable to therapy, either by targeting the underlying disease or by targeting downstream pathways that drive fibrosis [24,25]. Importantly, the elucidation of fibrogenic mechanisms at the molecular level has resulted in the identification of many rational therapeutic targets [24,25,26].

## 3. Extracellular Vesicles

### 3.1. EV Definition

**Figure 2:**
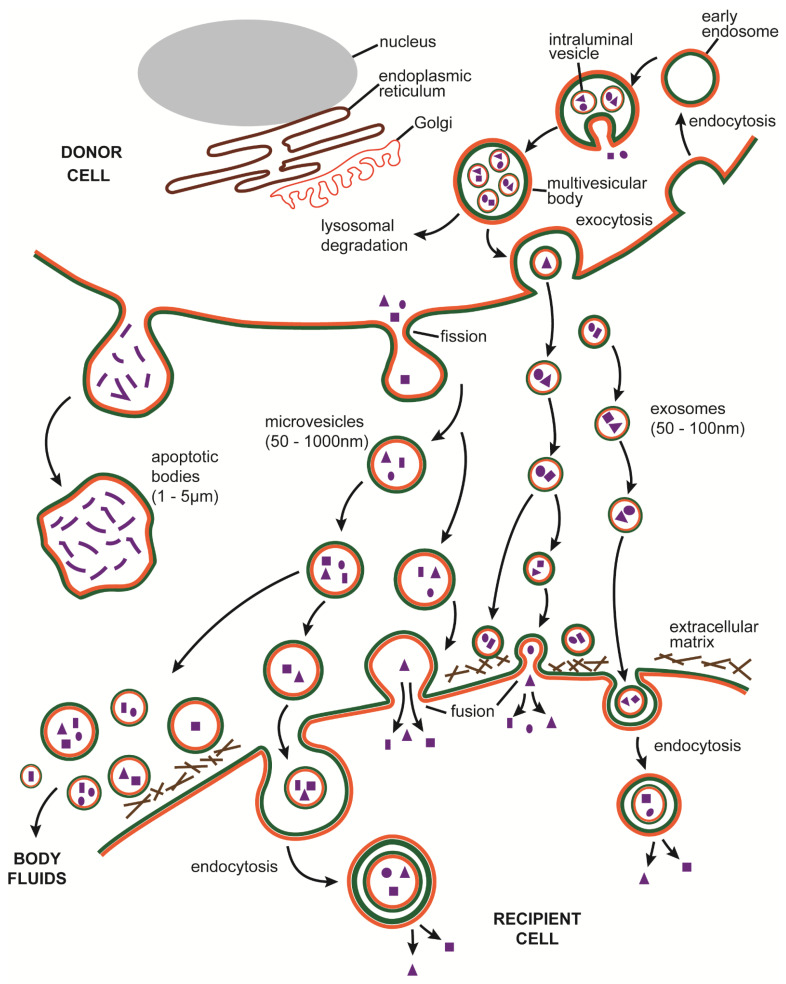
Pathways of EV biogenesis and action. Three type of EVs are produced by most cells. Exosome biogenesis is initiated by the involution and pinching off of the endosomal membrane resulting in the production of an ILV that contains cytoplasmic constituents. As ILVs accumulate, MVBs are generated, which are then either degraded via the lysosomal pathway or trafficked to the cell surface whereupon they fuse with plasma membrane and liberate their contents, now becoming exosomes, into the extracellular space. Mircovesicles also contain similar cytoplasmic constituents but are generated by fission of the plasma membrane. Apoptotic bodies contain components of cell degradation and form by cytoplasmic bulging and separation from the cell as a result of cytosketetal breakdown during cell disassembly. Microvesicles and exosomes may bind to ECM components in the interstitial space or may be internalized by target cells, either by fusion with the plasma membrane or by endocytosis, both of which result in delivery of their respective molecular payloads into the recipient cell. Once released into the extracellular space, EVs may alternatively be carried in interstitial fluids into the main body fluids allowing them to target cells at distant sites or to be cleared.

EVs are a heterogenous group of microscopic (<4–5 µm diameter) membrane-limited sacs that are released by many cell types and are found in interstitial spaces and body fluids. EVs are classified according to their mechanisms of biogenesis and currently comprise exosomes, microvesicles (microparticles), and apoptotic bodies. Exosome biogenesis is initiated by involution and fission of the endosomal membrane resulting in the production of intraluminal vesicles (ILV) within multivesicular bodies (MVBs), the latter of which then traverse to and fuse with the cell surface causing their internal vesicles to be released extracellularly as exosomes (Figure 2). Microvesicles are formed when the plasma membrane is “pinched off” by budding and fission and the resultant vesicles are shed extracellularly (Figure 2). Apoptotic bodies are also formed by outward budding of the plasma membrane, but the process is induced by self-destructive actions of the cytoskeleton during cell apoptosis (Figure 2). Apoptotic bodies, which are generally larger (1–5 µm) than other EVs, contain debris from dying cells and undergo phagocytosis by macrophages. In contrast, exosomes and microvesicles are smaller in size (50–100 nm and 50–1000 nm respectively) and carry a complex molecular cargo comprising proteins, mRNA, microRNA (miR), lipids, and metabolites, some or all of which can be delivered to other cells which may respond to some of the molecular information received. Since apoptotic bodies are not discussed in this review, the term “EV” is used herein to describe exosomes or microvesicles.

### 3.2. EV Biogenesis

Exosomes are, in part, generated by a pathway that is dependent on endosomal sorting complex required for transport (ESCRT) machinery. This involves the coordinated assimilation of ESCRT-0, -I, -II, and -III complexes from many individual proteins and these complexes then interact with accessory proteins to cause invagination and scission of endosomal membranes resulting in formation of MVBs [27,28,29,30]. The involvement of ESCRT proteins may result in them becoming actual exosome constituents and this is used experimentally (e.g., by Western blot) to invoke the involvement of the ESCRT pathway in their formation and hence their identity as exosomes: such proteins include tumor susceptibility gene 101 (TSG101), which is a component of ESCRT-1 or apoptosis-linked gene-2 interacting protein X, which is an ESCRT accessory protein [31]. An ESCRT-independent means of exosome production also exists, which involves uptake of ceramide into the endosomal membrane thereby increasing the lipid raft content and promoting inward membrane budding [28,29,30,32]. This process is dependent on neutral sphingomyelinase 2 (nSmase2), which catalyzes the production of ceramide from sphingomyelin and which can be blocked using nSMase2 inhibitors such as GW4869 or small interfering RNA [33,34,35,36,37,38]. Exosome biogenesis is also regulated by tetrapanins (e.g., CD9, CD63, CD81), which are involved in cargo selection and exosome release as well as by small Rab guanosine triphosphate hydrolases (GTPases), which control endosomal trafficking, fusion with the plasma membrane, and release from the cell [28,29,30,39,40]; tetraspanins and Rabs are often present in exosomes and are frequently used for EV characterization [31]. Importantly, ESCRT components and tetraspanins are also involved in the production of microvesicles even though the biogenic mechanism is quite distinct. Micovesicles are formed as a result of numerous changes in membrane protein composition and lipid reorganization, as well as the activation of flippases, floppases, scramblases, and cytoskeleton regulators (Rho GTPases, Rho-associated protein kinase (ROCK), ADP-ribosylation factor 1 and 6 (ARF1, ARF 6)) [30,41,42,43,44,45]. This results in membrane instability and bending, with microvesicles then being liberated from the cell surface by fission.

Discrimination between exosomes and microvesicles is experimentally challenging because they have overlapping sizes and discernment of the biogenic pathways involved is very difficult, especially after they have left their cells of origin. However, whereas certain proteins are common to all EVs (e.g., actin, ezrin, moesin, heat shock protein (HSP)-70, flotillin-1, MHC I and II), the presence or absence of specific tetraspanins (CD9, CD63, CD81), especially in combination with proteins associated with ILV/MVB (TSG101, syntenin-1) or plasma membranes, can help to discriminate exosomes from microvesicles [31,46,47]. Nonetheless, biological samples invariably contain a mixture of such EVs and unless extremely rigorous steps are adopted, the precise EV subpopulations in a given sample are extremely challenging to definitively ascertain.

### 3.3. EVs in the Biology of Fibrosis

Following their release from producer cells, EVs may influence the behavior and function of the same or other cells in the local vicinity. For example, EVs may either function as dynamic structural and functional components of the ECM and contextually regulate ECM structure, signaling, and cell behavior [48] or may fuse with or be endocytosed by target cells, which may then respond according to the molecular information received in the EV payload (Figure 2). EVs from disease sites often contain altered cargo components as compared to their heathy counterparts and these can elicit disease-related or pro-pathogenic responses in target cells; fibrosis is an example of a pathological process that is driven directly or indirectly by altered populations of EVs that are produced by damaged, invading, or activated cells during chronic injury. On the other hand, it has been possible to capitalize on the intrinsic cellular homing and reprogramming functions of EVs, coupled with the fact that they are immunologically inert, to develop EV-based therapeutics for a wide range of pathophysiological conditions, including fibrosis for which therapeutic options are very limited. EVs may be particularly suited to fibrosis therapy because their aquaporin-1-regulated hydration confers unique structural flexibility for potential penetration of fibrotic scar [49].

EVs are present in numerous body fluids (e.g., saliva, sputum, serum, plasma, uterine secretions, urine, bronchoalveolar lavage fluid (BALF)), which may either result in their removal from the body or allow them to exert their biological effects at locations distant from their site of production (Figure 2). EVs can be readily harvested from body fluids and in light of their diverse molecular cargo, they are a valuable component of “liquid biopsies” whereby quantitative or qualitative features of their constituent EV payloads may be used to aid disease diagnosis or prognosis and thus facilitate decisions regarding patient management and treatment [50]. In most chronic diseases, the severity of organ fibrosis is an important prognostic indicator, but it often can only be directly measured by biopsy which is invasive, risky, and not conducive to the multiple sequential determinations needed to establish fibrosis progression or regression. Harnessing the molecular information from EVs in body fluids is a non- or minimally-invasive option that favors repetitive sampling and builds upon a broad platform of potential biomarkers for assessment of fibrosis severity during disease advancement or treatment.

## 4. Pulmonary Fibrosis

### 4.1. Causes and Pathological Features of Pulmonary Fibrosis

In pulmonary fibrosis, lung parenchyma becomes replaced with scar resulting in hampered alveolar function, compromised gas exchange and decreased compliance resulting in hypoxia, shortness of breath, cough, wheezing, and fatigue [51,52]. Pulmonary fibrosis is caused by infections (e.g., tuberculosis), inhalation of particulates, scleroderma, or radiation therapy or may be idiopathic with unknown etiology. Idiopathic pulmonary fibrosis (IPF) is a chronic progressive inflammatory interstitial lung disease in which pulmonary function becomes irreversibly lost due to deposition of scar and thickening of the pleural lining, eventually leading to death [51,52,53,54]. One of the earliest events in IPF is reprograming or dysfunctioning of alveolar epithelial cells due to mutations, environmental factors, aging, senescence, death, depletion, or expression of genes that predispose to develop fibrosis. This is followed by inflammatory cell activation and infiltration and the local production of chemokines and cytokines (e.g., TGF-β) that drive epithelial-to mesenchymal transition (EMT), fibroblast differentiation and fibrocyte recruitment. This process culminates in the production and deposition of excess ECM proteins, increased matrix stiffness, and epigenetic reprogramming that drives profibrotic changes in fibroblasts and epithelial cells [55]. The anti-fibrotic drugs nintedanib and pirfenidone are indicated for slowing fibrosis progression in IPF but neither drug is curative and they are often used palliatively while awaiting lung transplantation [56].

Chronic occupational exposure of the lung to airborne particulates can cause severe inflammation and fibrosis resulting in shortness of breath, cough, fatigue, chest tightness, and cyanosis. For example, silicosis is caused by inhalation of crystalline silica particles which become deposited in the alveoli and surrounded by collagen resulting in diffuse nodular fibrosis [57,58]. In asbestosis, inhaled asbestos fibers become embedded deep in the lungs and trigger inflammation and interstitial fibrosis around the alveoli, restricting elasticity and gas exchange and predisposing to develop mesothelioma and lung cancer [58,59]. In these examples, macrophages are recruited to remove particulates or damaged airway epithelial cells but the ensuing inflammatory response is a trigger for fibrosis [58]. Exposure to cigarette smoke or other airborne pollutants can cause chronic obstructive pulmonary disease (COPD) which is typified by chronic bronchitis and/or emphysema and involves ECM remodeling due to altered interactions between polymorphonuclear leukocyte i.e., neutrophil (PMN)-secreted proteases and the antiprotease barrier [60,61]. PMN involvement is also a hallmark of bronchopulmonary dysplasia (BPD), which occurs in low birth weight premature infants receiving prolonged supplemental oxygen to treat respiratory distress syndrome [62,63]. Pathological features of BPD include necrotizing bronchiolitis, alveolar septal injury, inflammation, and fibrosis, although the necrotic and fibrotic components are usually avoided with surfactant therapy and high frequency ventilation [63].

The best characterized murine model of pulmonary fibrosis involves administration of the antibiotic bleomycin and this has been employed in most EV studies to date. Other pulmonary fibrosis models are based on administration of particulates or irradiation. In each case, there are certain clinical correlates, with the extent and timing of fibrosis being variable and influenced by the strain and species used [64,65].

### 4.2. Mechanistic Aspects of EVs in Pulmonary Fibrosis

#### 4.2.1. Production and Action of EVs from Lung Tissues or Pulmonary Fibroblasts

BALF from mice with bleomycin-induced pulmonary fibrosis or from IPF patients contained up to three times more EVs than BALF from controls [66]. EVs from both sources as well as EVs from human fibrotic lung explants or human lung fibroblasts contained increased levels of Wnt5A, a β-catenin-independent protein that was enhanced in EVs by TGF-β treatment of the EV producer fibroblasts. EVs from lung fibroblasts or BALF from IPF patients stimulated proliferation of fibroblasts in vitro and this was mediated at least partly by EV Wnt5A [66]. BALF-derived EVs from mouse bleomycin pulmonary fibrosis contained reduced concentrations of let-7d and this was associated with stimulation of pericyte transdifferentiation and fibrogenesis via enhanced action of its direct TGF-β receptor 1 (TGF-βR1) target and downstream *FoxM1/Smad/-β-catenin* signaling [67]. Further, EVs from fibrotic mouse or human lungs exacerbated experimental lung fibrosis in mice and caused transcriptomic changes or signaling in lung epithelial cells in vitro that were associated with fibroproliferation and fibrogenesis (e.g., TGF-β, Wnt/catenin etc.) [68]. Syndecan-1, which is required for fibroproliferative gene expression in alveolar type II cells, reduced levels of EV miRs that target components of TGF-β or Wnt/catenin signaling, thereby driving lung fibrosis in vivo [68]. In addition, EVs were proposed as a component of the senescent-associated secretory phenotype of senescent pulmonary cells that occurs in aging and heightens the chance of developing chronic lung diseases such as IPF and COPD [69]. Epithelial cell mitochondrial damage and senescence in lung epithelial cells was stimulated by EVs from IPF lung fibroblasts and attributed to suppression of *SIRnotch3* by EV miR-23b-3p and miR-494-3p, the levels of which were correlated with IPF disease severity [70]. Finally, EVs from TGF-β-stimulated MRC5 human lung fibroblasts contained enhanced levels of programmed death-ligand 1 (PD-L1) that contributed to the ability of the EVs to decrease T cell proliferation and increase MRC5 cell migration and which suggest a role for EV immune checkpoint proteins in pulmonary immunosuppression and fibrosis [71].

#### 4.2.2. Production and Action of EVs from Lung Epithelial Cells or Macrophages

Exosomes from cultured M2 macrophages or macrophages from rat bleomycin pulmonary fibrosis caused increased proliferation and expression of *collagen 1A/3A* or *αSMA* in cultured pulmonary interstitial fibroblasts and this was attributed to targeting of *FAM13A* by miR-328 [72]. In bleomycin pulmonary fibrosis, *collagen 1* and *αSMA* levels were attenuated by administration of either miR-328-depleted M2 macrophages or their exosomes [72]. Lipopolysaccharide (LPS)-induced acute lung injury in mice resulted in the presence of macrophage-derived exosomes in BALF that were enriched in pro-inflammatory tumor necrosis factor-α (TNF-α), which was proposed to activate neutrophils from which 1L-10-enriched exosomes were then released causing polarization of macrophages to the M2 phenotype and downstream fibrotic sequala [73]. The proteomic content of exosomes from lung epithelial or macrophage cell lines was qualitatively and quantitatively changed when the cells were exposed to asbestos and these exosomes caused expression of mesenchyme- and cancer-related genes in mesothelial cells, which were consistent with a transition to a mesothelioma-related phenotype [74,75]. Exosomes from silica-exposed RAW264.7 macrophages stimulated αSMA production in fibroblasts and contained differentially expressed miRs that had predicted effects on cell differentiation, proliferation, and collagen production, and which targeted the TGF-β pathway [76]. MiR-125a-5p was one such component since it was up-regulated in EVs from silica-treated macrophages and it induced TGF-β-mediated fibroblast transdifferentiation by downregulating *Smurf1*. Levels of serum exosomal miR-125a-5p were higher in patients with silicosis as compared to healthy controls [76]. Bleomycin-induced pulmonary fibrosis was associated with increased serum levels of exosomal miR-22 and fibrotic pathology was ameliorated with a miR-22 mimic due to its ability to inhibit TGF-β1-induced expression of *αSMA* or profibrotic cell communication network factor 2 (*CCN2*; also known as connective tissue growth factor) in lung fibroblasts in vitro [77].

#### 4.2.3. Production and Action of EVs from Endothelial Cells

Exosomes from pulmonary microvascular endothelial cells contained miR-107, which inhibited hypoxia inducible factor-1α (*HIF-1α*) in pericytes, resulting in suppression of a Notch1/PDGFRβ/yes associated protein 1 (YAP1)/Twist1 axis and downstream inhibition of *αSMA* and *collagen 1α1* expression [78]. During pulmonary fibrosis, endothelial cell EV miR-107 levels were downregulated, resulting in enhanced *HIF-1α* expression and stimulation of pericyte transdifferentiation and fibrogenesis [78].

**Figure 3:**
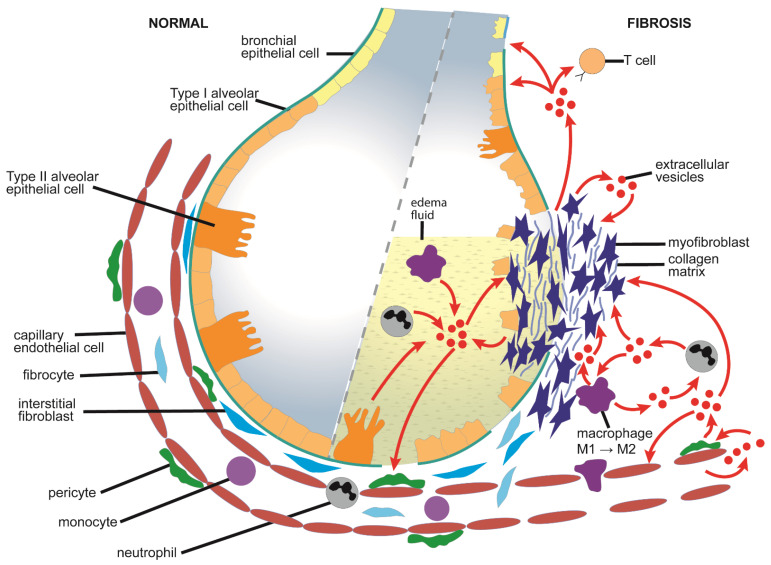
Proposed EV pathways in the pathogenesis of pulmonary fibrosis. Prolonged alveolar injury results in the production and interstitial deposition of type I collagen by αSMA-positive myofibroblasts and the resulting increased edema and expansion of fibrotic ECM impinges on alveolar air space, structure and function, severely limiting gaseous exchange and decreasing lung performance. Myofibroblast expansion is the result of transdifferentiation/activation of resident interstitial fibroblasts, bone marrow-derived fibrocytes, endothelial cells, epithelial cells, or pericytes. Altered EV components such as Wnt5a and let-7d from these cell types are proposed to promote the transition into myofibroblasts and/or to enhance activation and fibrogenesis in the accumulating myofibroblast population through the regulation of TGF-β, Smad and β-catenin pathways, while increased EV PD-L1 dampens T cell responses and promotes fibroblast migration. Further, miR-23b-3p and miR-494-3p in EVs from activated fibroblasts suppress notch signaling and drives cell senescence in epithelial cells. EVs from infiltrating or activated M2 macrophages contain altered levels of miRs-328 and -125a-5p which drive fibroblast transdifferentiation and collagen production in fibroblasts, and this process is exacerbated by the effect of 1L-10-enriched EVs from TNF-α-primed neutrophils. Pro-fibrogenic EVs in BALF originate from damaged, infiltrating, and activated cells in lung tissue and edema fluid but in most studies the precise cellular sources of BALF EVs have not been definitively determined. Transdifferentiation of pericytes is triggered as a response to EVs from BALF or capillary endothelial cells which contain, respectively, suppressed let-7d or miR-107. Only cells with a demonstrated role in EV production or response are shown; some of the depicted EV pathways are surmised from in vitro observations and have not been demonstrated in vivo. See text for details.

Some of the pathways of EV-regulated pulmonary fibrosis discussed above are shown in Figure 3.

### 4.3. Therapeutic Actions of EVs in Pulmonary Fibrosis

#### 4.3.1. EVs from Adult Stem Cells

Exosomes from human bone marrow mesenchymal stem cells (BM-MSC) prevented or reversed bleomycin-induced pulmonary fibrosis in mice by improving pulmonary morphology and decreasing collagen deposition [79]. Therapy was associated with a switch from pro-inflammatory lung monocytes and macrophages to homeostatic populations (e.g., non-classical monocytes) present in control animals. Concomitant changes occurred in the bone marrow myeloid cell population that were consistent with proteomic changes induced by exosome treatment of monocytes in vitro and, further, i.v. administration of bone-marrow-derived monocytes that had been pre-treated with BM-MSC exosomes in vitro prevented bleomycin pulmonary fibrosis [79]. Administration of EVs from BM-MSC or umbilical cord Wharton’s jelly MSC (WJ-MSC) to a mouse BPD model normalized the expression of hyperoxia-sensitive genes involved in immunity and inflammation and suppressed proinflammatory M1 macrophages while enhancing anti-inflammatory M2 macrophages [80]. This was accompanied by EV-mediated improvement of lung architecture and function, reduced pulmonary inflammation and fibrosis, and rescue of peripheral vascular deficits [80] as well as reversal of long-term pulmonary complications (e.g., pulmonary hypertension, exercise capacity etc.) [81]. EVs from WJ-MSC were also protective in a rat model of monocrotaline-induced pulmonary hypertension, resulting in restored cardiac function and attenuation of pulmonary fibrosis and vascular remodelling, the latter being attributed to EV-regulated Wnt5a/bone morphogenic protein (BMP) signaling in vascular smooth muscle cells and endothelial cells [82].

In vitro, activation of LL29 pulmonary fibroblasts was suppressed by BM-MSC EVs and this was due to targeting of frizzled class receptor 6 (*FZD6*) by EV miR-29b-3p, which was also shown to be required for the inhibition by BM-MSC EVs of bleomycin-induced pulmonary fibrosis in mice [83]. Exposure of LPS-treated MLE-12 type II alveolar epithelial cells to BM-MSC exosomes resulted in inactivation of the nuclear factor kappa-light-chain-enhancer of activated B cells (NF-κB) pathway, reversal of EMT due to targeting of *Ikbkb* by exosomal miR-182-5b, and ubiquitinylation of Ikbkb due to targeting of *Usp5* by exosomal miR-23a-3p [84]. In a mouse model of silicosis, lung dysfunction and fibrosis were reduced by exosomes from human umbilical cord MSC (UMSC), which were also effective in decreasing collagen deposition in silica-exposed fibroblasts in vitro [85]. In radiation-induced lung injury in mice, the senescence-associated secretory phenotype, endothelial damage, inflammation, and fibrosis were reduced by placenta-derived MSC EVs, with therapy being attributed partly to EV miR-214-3p, which targeted ataxia telangiectasia mutated (*ATM*) to reduce ATM/P53/P21 signaling and DNA damage [86].

In a rat model of lung injury and fibrosis caused by intratracheal instillation of ≤2 µm particulates, administration of adipose-derived MSC (AD-MSC) EVs resulted in restoration of lung architecture, reduced apoptosis and necrosis in type II alveolar cells, and suppression of reactive oxygen species (ROS), inflammation and fibrosis, the latter of which was associated with targeting of *TGF-βR1* by EV let-7d-5p [87].

Amnion epithelial cells (AECs) have a high capability for multipotent differentiation and can be induced to exhibit lung lineage–specific markers and to develop into differentiated lung cells such as type II alveolar pneumocytes. In models of ovalbumin/napthalene-induced allergic airways disease or bleomycin-induced pulmonary fibrosis, intranasal administration of AEC exosomes resulted in reduced inflammation, epithelial damage, ECM, myofibroblast frequency, collagen content, fibrosis, and/or TGF-β expression [88,89]. These outcomes were associated with normalized airway reactivity and dynamic lung compliance and were augmented by co-administration of serelaxin (recombinant human relaxin-2) [88]. AEC exosomes contained proteins related to apoptosis, development, mitogen-activated protein kinase (MAPK), inflammation, and growth factor signaling and miRs predicted to impact pathways of fibrosis, cancer and stem cell pluripotency [89]. In vitro, AEC exosomes lowered neutrophil survival and myeloperoxidase activity, suppressed T cell proliferation and stimulated macrophage phagocytosis and their anti-inflammatory M2 phenotype [89].

Cultured lung tissue gives rise to self-aggregating three-dimensional spheroids comprising progenitor cells and supporting stromal cells. In rodent models of silica- or bleomycin-induced pulmonary fibrosis, administration of a nebulized lung spheroid cell secretome or its constituent exosomes decreased alveolar epithelial damage, reduced collagen deposition, decreased vascular injury, and improved pulmonary function, with miR-30a and the let-7 and mirR-99 families being implicated in the therapeutic effects [90].

Administration of EVs from menstrual blood-derived stems cells MenSC to mouse bleomycin pulmonary fibrosis models resulted in reduced alveolar epithelial cell injury, reduced pulmonary fibrosis, and restoration of hyroxyproline, malondialdehye, and glutathione peroxidase to baseline values [91]. The therapeutic activity was associated with EV-mediated delivery into alveolar epithelial cells of let-7, which suppressed production of lectin-like oxidized low-density lipoprotein scavenger receptor-1 (*LOX1*) and downstream NLR family pyrin domain containing 3 (NLRP3)-mediated apoptosis [91].

#### 4.3.2. EVs from Lung Fibroblasts or Macrophages

EVs produced by lung fibroblasts from IPF patients induced senescence in lung epithelial cells via EV-stimulated mitochondrial ROS and DNA damage response activation, and these effects were due to the targeting of surtuin-3 (*SIRT-3*) by elevated EV levels of miR-23b-3p and miR-494-3p [70]. Expression of *TGF-βR1* and fibrotic genes in alveolar epithelial cells or lung fibroblasts was suppressed by miR-142-3p in EVs from THP1 macrophages, supporting a potential anti-fibrotic role for this miR, which was enriched in in EVs from IPF sputum macrophages [92].

#### 4.3.3. EVs from Serum

Acute respiratory distress syndrome (ARDS) is characterized by rapid-onset pulmonary inflammation and edema that results in death of 33% of affected individuals and a high incidence of interstitial and alveolar fibrosis in survivors. Exosomes from the serum of patients with ARDS contained reduced levels of miR-425, which targets the lysine demethylase 6A-TGF-β-Smad axis in lung fibroblasts [93]. Suppression of miR-425 expression in lung fibroblasts led to their enhanced proliferation and production of collagens, suggesting that fibrotic pathways in ARDS are regulated by cellular or exosomal miR-425 [93]. EVs from serum of mice with bleomycin-induced pulmonary fibrosis contained increased levels of several miRs including miR-22 [77] and miR-16 [94]. Bleomycin-induced pulmonary fibrosis was attenuated by mimics of either miR-22 or miR-16 which, when tested on TGF-β-treated lung fibroblasts in vitro, caused suppressed expression of, respectively, *αSMA* and *CCN2* [77] or rapamycin-insensitive companion of mechanistic target of rapamycin (mTOR) (*Rictor*) and secreted protein acidic and rich in cysteine (*Sparc*) [94].

### 4.4. EVs as Biomarkers for Pulmonary Fibrosis

Serum EV miR-21-5p was significantly elevated in the acute inflammatory phase and chronic fibrotic phase in mouse bleomycin lung fibrosis and was correlated with decreased vital capacity and survival in IPF patients [95]. In IPF patients, sputum exosomal miR-142-3p and miR-33a-5p were increased while let-7d-5p was decreased, with miR-142-3p and let-7d-5p being respectively, inversely or positively associated with severity of lung dysfunction [96]. Some differentially expressed sputum exosomal miRs were predicted to have targets related to inflammation, ECM/collagen production and EMT [96]. IPF disease severity was positively corelated with levels of EV miR-23b-3p and miR-494-3p from the patients’ lung fibroblasts [70]. MiR-142-3p was significantly upregulated in EVs of IPF sputum and plasma and was strongly associated with macrophage frequency [92].

## 5. Renal Fibrosis

### 5.1. Causes and Pathological Features of Renal Fibrosis

Renal fibrosis is a hallmark of numerous chronic kidney diseases (CKD), which affect millions of people globally [97]. CKD patients have an irreversible deterioration of renal function, which results in end-stage renal failure for which the only treatments are kidney transplantation or hemodialysis. Glomerulosclerosis and tubulointerstitial fibrosis are triggered by sustained injury or stress to cells of the glomerulus or collecting tubule due to insults that are metabolic, immunological, toxic, or mechanical [98,99]. Scarring impedes the critical interactions between cells of the tubules, glomerulus, and capillaries resulting in compromised filtration and resorption, and a progressive loss of renal function [100,101,102]. The severity of renal fibrosis is highly correlated with progression of CKD and is a major contributor to CKD pathophysiology.

The most common cause of end-stage CKD is diabetic nephropathy (DN), a microvascular complication of diabetes mellitus involving hemodynamic changes and oxidative stress caused by hypergycemia [103]. This results in the production of ROS, cell damage, release of pro-inflammatory cytokines, recruitment of inflammatory cells, mesangial cell expansion, glomerulosclerosis, and fibrosis [104]. Glomerular injury is a hallmark of early DN and involves macrophage infiltration, thickening of basement membranes, mesangial expansion, and loss of glomerular cells leading to breakdown of the glomerular filtration barrier, which is manifested clinically as proteinuria. Tubular hypertrophy and interstitial inflammation occur in the early phases of DN and is followed by tubular atrophy and interstitial fibrosis as the disease progresses [105]. Progression of DN is slowed using medications to control hypergycemia or hypertension but there is an urgent need for therapies that act directly on renal fibrosis itself. CKD is also caused by acute kidney injury (AKI), such as that due to hypoxia or ischemia/reperfusion (I/R) [106]. AKI is associated with a high morbidity and mortality and involves sudden kidney damage that occurs over hours or days. While AKI is self-limiting in some individuals, there is no treatment and it is a major risk factor for CKD because the acute insult leaves the kidneys permanently injured or exacerbates chronic renal failure [107].

Several in vivo EV studies have utilized the unilateral ureteral obstruction (UUO) model in mice or rats, which reliably results in rapid and severe tubulo-interstitial renal fibrosis. Other EV investigations have been undertaken in injury or disease models in which renal fibrosis is a common pathological feature such as I/R, 5/6 nephrectomy, streptozotocin (stz)-induced diabetes mellitus, and aristolochic acid nephropathy (AAN) [100].

### 5.2. Mechanistic Aspects of EVs in Renal Fibrosis

#### 5.2.1. Production and Action of EVs from Epithelial Cells

Renal fibrosis in vivo was shown to be EV-dependent by treating mice with the exosome inhibitors GW4869 or dimethyl amiloride (DMA) which resulted in decreased fibrosis and decreased expression of fibronectin (FN), αSMA, collagen I, and/or fibroblast-specific protein in I/R or UUO models [108,109]. EVs produced by the I/R kidney were enriched in miR-150, which was directly fibrogenic in its mimic form, while EVs from NRK-52E proximal tubular epithelial cells (TEC) were stimulatory or inhibitory for I/R kidney fibrosis when they were respectively, enriched or depleted of miR-150 [108]. In vitro, cellular and EV levels of miR-150 were enhanced in NRK-52E cells under hypoxic conditions and its EV-mediated delivery to NRK-49F fibroblasts resulted in their enhanced proliferation and suppressed apoptosis [108]. Similar pathways, in which TEC exposed to stress, injury, or pro-fibrogenic molecules (e.g., TGF-β) produce EVs that are functionally delivered to other TECs or fibroblasts, have been documented in several other studies. For example, EVs from TGF-β-stimulated NRK-52E cells stimulated mesenchymal transition in recipient TECs and this was mediated by EV miR-21, which targeted phosphatase and tensin homolog (*PTEN*) and enhanced protein kinase B (AKT) signaling in the recipient cells [110]. EVs from high glucose treated mouse proximal TEC cells induced proliferation and production of *FN*, *αSMA*, and *collagen I* in NRK-49F rat kidney interstitial fibroblasts [111]. Human proximal TEC exposed to high glucose became senescent and liberated miR-21-containing EVs that promoted subsequent EMT in the cells through its suppression of peroxisome proliferator-activated receptor α (PPARα) and enhanced HIF-1 signaling supporting a role for miR-21 in age-related fibrosis [112]. Exposure of TEC to hypoxia in vitro caused their exosome production to increase and these exosomes (unlike their normoxic counterparts) promoted proliferation and enhanced levels of *TGF-β1*, *αSMA*, *F-actin*, and *collagen α1* in renal tubulointerstitial fibroblasts [113]. The activation-inducing property was dependent on exosomal *TGF-β* mRNA, the level of which was also elevated in exosomes produced during hypoxic fibrosis in the UUO model in mice [113]. Similar data were reported in the UUO model, while renal exosome production in vivo was enhanced after I/R injury or 5/6 nephrectomy [109]. Expression of sonic hedgehog (*Shh*) was associated with exosome production in vivo and was also required for exosome production by TGF-β-treated HKC-9 kidney proximal TEC in vitro. Moreover, exosomes from TGF-β-treated HKC-9 cells activated fibroblasts in vitro and exacerbated kidney injury and fibrosis in vivo, the latter of which was *Shh*-dependent [109]. Further, cellular export by TEC of transglutaminase 2 (which stimulates ECM cross-linking and functions during fibrosis progression) occurred through its incorporation into TEC EVs, production of which was stimulated by TGF-β [114]. Upon EVs being released from their producer TEC, transglutaminase 2 promoted fibrogenic pathways via direct binding interactions with ECM proteins and stimulation of myofibroblast functions [114]. Aldosterone-induced renal fibrosis in *db/db* mice involved export of EV miR-196b-5p from TEC and its delivery into fibroblasts resulting in downregulation of suppressor of cytokine signaling 2 (SOCS2) expression and enhanced expression of signal transducer and activator of transcription 3 (*STAT3*), *FN*, *αSMA*, *or collagen1α1* [115].

#### 5.2.2. Production and Action of EVs from Podocytes, Endothelial Cells, Mesangial Cells, or Macrophages

Podocytes are terminally differentiated epithelial cells that interact with TEC or glomerular cells to maintain renal function but which become functionally compromised and depleted in DN resulting in proteinuria and glomerulosclerosis. Podocyte exosomes are present in human urine [116] and may target TEC since in vitro addition of EVs from human differentiated podocytes to human proximal TEC caused p38-MPAK-dependent Smad3 phosphorylation and activation of TGF-βR1, resulting in enhanced expression of *FN* and *collagen IV* [117]. Podocytes undergo EMT during DN, which further compromises glomerular function. Podocytes were injured by EVs from glomerular endothelial cells (GEC) that had undergone endothelial-to-mesenchymal transition after exposure to high glucose as occurs in DN [118]. In vitro, EVs were produced at higher frequency by glucose-treated GEC and the EVs both promoted mesenchymal transition in podocytes by a process involving *Wnt/β-catenin* signaling [118] and stimulated activation of glomerular mesangial cells (GMC) by mechanisms that involve EV TGF-β levels [119] and EV delivery of PI3K/AKT- and MAPK-associated circular RNAs [120]. High glucose-treatment of GMC caused EVs to be produced at higher frequency and they caused injury in podocytes (increased apoptosis and TGF-β1/PI3-AKT signaling; reduced cell adhesion and expression of nephrin, podocin, and WT-1 [121] while stimulating fibrosis-related changes in control GMC (increased production of FN, angiotensin II (Ang II), renin, AT_1_/AT_2_) [122]. Finally, high glucose-treated RAW264.7 macrophages stimulated activation of mouse GMC via *TGF-β1/Smad* signaling [123] and promoted activation, proliferation, and inflammatory cytokine production in control RAW264.7 macrophages [124].

**Figure 4:**
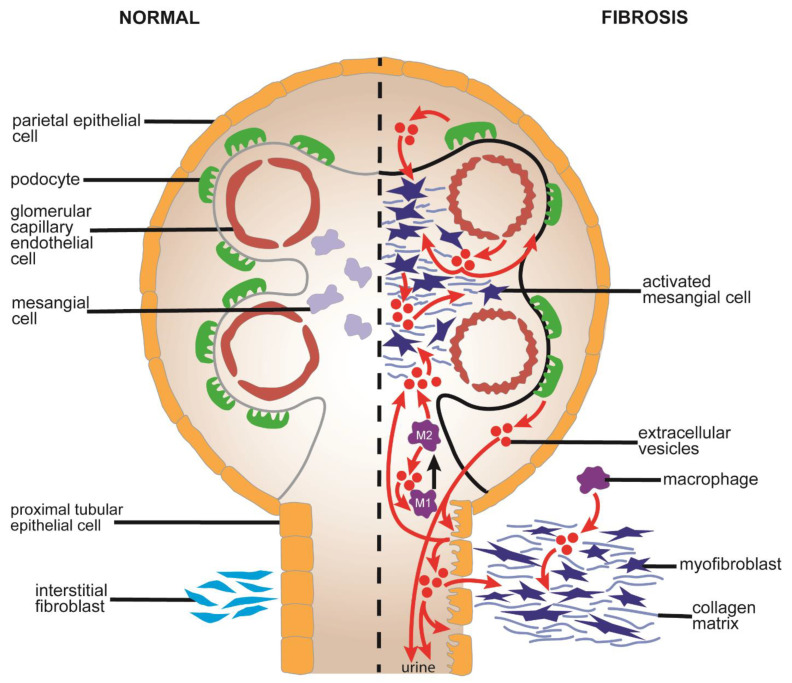
Proposed EV pathways in the pathogenesis of renal fibrosis. EVs are produced in enhanced numbers by stressed or injured TEC downstream of *Shh* and they cause proliferation and fibrogenesis in fibroblasts, in part by downregulation of *SOCS2* by EV miR-196b-5p. TEC EVs also drive EMT and activation in TEC themselves via processes that are dependent on the action of EV TGF-β, transglutaminase 2, or miR-21, the latter of which is associated with regulation of PTEN/AKT or PPAR/HIF in recipient cells. Podocyte injury and drop-out is a feature of DN and EVs from these cells are present in urine and, when produced in the context of DN-like high glucose conditions, drive ECM production via a TGF-β/p38/Smad axis in TEC and mesangial cells. High glucose levels also result in the production of EVs by GEC that drive Wnt/β-catenin-mediated EMT in podocytes or circular RNA-mediated EMT in mesangial cells. Macrophages exposed to high glucose produce pro-inflammatory EVs that are themselves macrophage-activating and further stimulate TGF-β-dependent activation of mesangial cells. Production by mesangial cells of pro-fibrogenic and pro-pathogenic molecules such as FN and Ang II is stimulated by EVs that are produced by mesangial cells exposed to high glucose concentrations. Only cells with a demonstrated role in EV production or response are shown; some of the depicted EV pathways are deduced from in vitro observations and have not been demonstrated in vivo. See text for details.

Some of the pathways of EV-regulated renal fibrosis discussed above are shown in Figure 4.

### 5.3. Therapeutic Actions of EVs in Renal Fibrosis

#### 5.3.1. EVs from Adult Stem Cells

In a rat model of stz-induced diabetes mellitus, administration of BM-MSC exosomes reversed renal tubule expansion, inflammation, interstitial fibrosis, cellular atrophy, and expression of *TGF-β* in TEC [125]. Furthermore, primary TEC cultures derived from stz-induced diabetic rats exhibited reduced degeneration and atrophy when treated with BM-MSC exosomes [125] while BM-MSC exosomes reduced stz-induced DN with improved renal function and structure and decreased expression of mTOR and fibrosis-related gene expression. The therapeutic mechanism appeared to involve autophagy (degradation/recycling of proteins/organelles to maintain homeostasis) as exosomes were less effective in the presence of autophagy inhibitors [126]. Likewise, in DN mice, human BM-MSC exosomes corrected kidney dysfunction and inhibited onset and progression of glomerular and interstitial fibrosis, with the anti-fibrotic effects being attributed in part to anti-fibrotic EV miRs [127]. BM-MSC EVs also attenuated renal fibrosis, inflammation, oxidative stress, and apoptosis in TGF-β-stimulated HK-2 proximal TECs or a UUO rat model, with the therapeutic effects being attributed to inhibition of *Rho/ROCK* by EV-mediated delivery of milk fat globule-epidermal growth factor-factor 8 (MFG-E8) [128]. BM-MSC EVs from older donors showed reduced therapeutic efficacy and was associated with reduced EV levels of miR-133 and -294 [129].

Administration of let-7c-enriched exosomes in the UUO model caused enhanced renal let7c expression, decreased kidney injury, and suppressed expression of collagen IV, matrix metalloprotease-9 (MMP-9), TGF-β1, or TGF-βR1 [130]. Further, let7c-enriched MSC exosomes reduced TGF-β-mediated expression of fibrotic genes in NRK cells in vitro and this was associated with down-regulation of *TGF-βR1* [130]. Similarly, in TGF-β-treated HK-2 TEC, the inhibited expression of *TJP1* or *E-cadherin* and stimulated expression of *αSMA*, *FN*, *Notch-1* or *Jagged-1* were restored to control levels by miR-34a-enriched BM-MSC EVs suggesting that the inhibition by of EMT by EV miR-34a was due to targeting of the *Jagged-1/Notch-1* pathway [131].

AD-MSC exosomes were therapeutic in a pig model of metabolic syndrome and renal artery stenosis in which improved kidney effects included reduced inflammation and fibrosis and appeared to be dependent on exosomal interleukin (IL)-10 [132]. Moreover, as compared to AD-MSC EVs from pigs with metabolic syndrome, those from lean pigs were more effective in improving renal function and decreasing inflammation and fibrosis in the stenotic kidney [133,134]. The therapeutic effects were associated with an enhanced M2:M1 ratio and frequency of CD8+ T cells possibly driven by anti-inflammatory actions of EV TGF-β as well as decreased oxidative stress due to EV mitochondria-regulating miRs [133,134]. In a deoxycorticosterone acetate (DOCA)-salt hypertensive model, AD-MSC EVs were therapeutic both in the kidney, in which filtration rate, fibrosis inflammatory reactions were attenuated, and in the cardiovascular system, as shown by attenuation of cardiac fibrosis and normalization of blood pressure [135]. The kidney effects were attributed to EV-mediated regulation of a miR200-TGF-β axis, which damped EMT [135]. EVs isolated from human AD-MSC that had been transfected with glial cell line-derived neurotrophic factor reduced peritubular capillary loss and fibrosis in the mouse UUO model and enhanced migration and angiogenesis and reduced apoptosis in HEVECS in vitro [108]. The endothelial benefit was due to EV-enhancement of SIRT-1 signaling and levels of phosphorylated endothelial nitric oxide synthase, which was proposed to result in post-injury angiogenesis and to contribute to reduced renal fibrosis [108].

In the UUO model in rats, EVs from human UMSC attenuated renal injury and interstitial fibrosis while promoting proliferation but suppressing oxidative stress and apoptosis in renal TEC, the latter of which was attributed to inhibition of ROS-mediated activation of p38MAPK/extracellular signal-regulated kinase (ERK) signaling [136]. It was further shown that the anti-fibrotic effect in this model involved EV-mediated delivery of casein kinase 1δ and E3 ubiquitin ligase β-TRCP resulting in ubiquitinylation and degradation of YAP, thereby attenuating YAP activation and collagen production [137]. Human UMSC EVs were therapeutic for kidney injury and fibrosis in models of renal ischemia and this outcome was augmented by EV Oct-4, which reduced EMT by inhibiting expression of *Snail* [138,139]. Finally, EV-mediated mechanisms for the inhibition by UMSC of inflammation and fibrosis in stz-induced DN in rodents was supported by the findings that the production of *TGF-β*, *IL-6*, *IL-1β*, and *TNF-α* in high glucose-injured HK2 human proximal TEC was inhibited by conditioned medium or EVs from UMSC [140] and that the removal of EVs from UMSC conditioned medium impaired its ability promote promoting proliferation or expression of *FN*, *MMPs*, or *collagen 1* in high glucose-cultured mesangial cells [141].

In a model of AAN, EVs from BM-MSC or human liver stem cells attenuated tubular necrosis, interstitial fibrosis and cellular infiltration and involved EV-mediated changes in fibrosis-, inflammation- and/or apoptosis-related mRNA and miR levels in the diseased kidneys [138,139,142,143]. Administration of placental MSC (PMSC) EVs in the UUO model resulted in decreased αSMA and collagen-I staining and increased infiltration of Foxp3+/IL-17+ CD4+ T cells, mimicking some of the anti-fibrotic and anti-inflammatory properties directly exhibited by PMSC in the same model [144]. Further, in an in vivo model of Ang II -induced cardiac hypertension and concomitant chronic kidney injury, renal function, inflammation, and fibrosis were attenuated by administration of cardiosphere-derived cells (CDC)-exosomes, the effect of which was attributed to exosomal Y RNA fragment, *EV-YF1*, which modulated IL-10 levels [145].

#### 5.3.2. EVs from Differentiated Cells or Urine

In AKI rat models employing renal ischemia/hypoxia, i.v. injection of exosomes from normoxic rat TEC resulted in improved renal function and reversed tubular damage, neutrophil fibrosis, vascular alterations, and transcriptomic changes [146]. Similar results occurred in a nude rat model of hypoxic renal injury after administration of exosomes from human TEC [147]. Interestingly, EVs from hypoxia-conditioned TEC were also therapeutic, sometimes even more so than their normoxic counterparts [146] which is enigmatic considering that EVs from damaged or injured TECs are pro-pathogenic [109,110,113,114]. In HEK 293 epithelial cells, a micellized form of BMP-7 was endosomally internalized (rather than binding its cell surface receptors), resulting in antagonism of TGF-β-induced EMT and the production of EVs containing active BMP-7 and these combined effects were proposed to contribute to the anti-fibrotic effect of micellized BMP-7 in a pig UUO model [148].

Several lines of evidence suggest that miR-29a is an important antifibrotic agent in the kidney. Expression of miR-29a was reduced in the kidney in rodent UUO models [149] and in urinary exosomes from CKD patients [150,151,152], while renal fibrosis was suppressed after administration of AAV-miR-29 in mice with UUO or DN [153]. Muscle satellite cell exosomes engineered to contain enriched levels of miR-29a and to elaborate a surface RVG-moiety that targeted acetylcholine receptors in the kidney were therapeutic after intramuscular injection in the UUO model [154]. These exosomes reversed muscle wasting and partially suppressed renal fibrosis and fibrosis-related gene expression, with the therapeutic effects of miR-29a in muscle or kidney being attributed to direct inhibition of, respectively *YY1* or *TGF-β3* [154]. Similar results were obtained for HEK293 exosomes enriched in miR-26a, which suppressed renal fibrosis in UUO kidneys by targeting pro-fibrotic CCN2 [155].

### 5.4. EVs as Biomarkers in Renal Fibrosis

EVs in urine are particularly attractive as a source of molecular information that can be harvested non-invasively and assessed for renal fibrosis-related biomarkers. Several studies have shown that the amount of miR-29c in urinary exosomes from patients with CKD and renal interstitial fibrosis is markedly reduced and correlated with renal function and degree of histological fibrosis [150,151,152]. Similarly, in lupus nephritis, declining urinary exosomal miR-29c levels were negatively correlated with early renal fibrosis, degree of histological chronicity and exosomal *Smad3* and *MMP2* mRNA levels [156], urinary EV miR-146a was related to disease activity and was associated with *TRAF6* suppression in driving inflammation and fibrosis [157], while urinary EV miR-31, -107 and -135-b are predictive of renal recovery and collectively target fibrosis-related *HIF-1α* [158]. Other studies of CKD showed that levels in urinary exosomal *CD2AP* mRNA (a podocyte molecule), miR-21, miR-181a, E-cadherin, or vimentin were correlated with severity of tubulointerstitial fibrosis and glomerulosclerosis [150,151,152,156,159,160]. Levels of vitronectin in urine and urinary EVs [161] or of miR-21 in plasma EVs [162] were proposed as biomarkers for post-kidney transplantation patients with a high incidence of tubulointerstitial fibrosis. Finally, miR-29a was elevated in serum EVs from mice with UUO [153] and plasma-derived exosomal miRs in rats that had undergone 5/6th partial nephrectomy or two-kidney-one-clip were associated with pathways of fibrosis and injury and could be discriminated from free plasma miRs [163].

## 6. Cardiac Fibrosis

### 6.1. Causes and Pathological Features of Cardiac Fibrosis

Although therapies for heart disease have improved significantly, a huge medical and financial burden remains and additional treatment strategies are needed. One promising approach is to target cardiac fibrosis because this pathological feature is widespread, but it is especially prevalent after myocardial infarction (MI) or during heart disease [164]. In MI, blockage of the coronary artery leads to cardiomyocyte death and damage and functional muscle mass is replaced with proliferative collagen-producing myofibroblasts that arise from transdifferentiation of cardiac fibroblasts, which comprise ~75% of all cardiac cells [164,165,166]. As myocytes are lost and fibrotic scar is deposited, the ventricle undergoes remodeling (myocyte hypertrophy and elongation, increased wall mass, chamber enlargement) to maintain cardiac output but ventricular structure and performance continue to decline and heart failure is inevitable. In the systolic form of heart failure, the replacement of cardiomyocytes by fibrotic scar results in ventricular stiffness that causes reduced ejection fraction (reduced cardiac output and systemic perfusion capacity), while in the diastolic form of heart failure, the ejection fraction is unchanged but the presence of fibrosis causes blood filling during diastole to be reduced [167].

There are several well-characterized animal models of heart failure and associated inflammation and fibrosis [168,169] but EV studies of cardiac fibrosis have principally used animal models of MI, with or without perfusion.

### 6.2. Mechanistic Aspects of EVs in Cardiac Fibrosis

#### 6.2.1. Production and Action of EVs from Cardiomyocytes

Primary cardiomyocyte cultures exposed to hypoxia or angiotensin II (Ang II) produced miR-208-enriched exosomes that promoted miR-208a-dependent myofibroblastic transition in cardiac fibroblasts [170]. Exosomes from hypoxic cardiomyocytes exacerbated cardiac dysfunction, cardiac fibrosis, and myofibroblast differentiation in post-MI rats and caused cardiac miR-208a levels to be increased, with post-MI cardiac dysfunction and fibrosis being driven by miR-208-mediated targeting of the differentiation-regulating factor *Dyrk2* [170]. Cardiomyocytes exposed to ischemia and reoxygenation in vitro produced EVs that elicited profibrotic gene expression in fibroblasts and were enriched in long noncoding (lnc) RNAs [171]. *Neat 1*, a lncRNA which predominates in large EVs, was required for in vitro cardiomyocyte or fibroblast survival and also reduced migration, cell cycle progression, and fibrotic gene expression in fibroblasts. In vivo, *Neat 1* was induced shortly after onset of MI in mouse models and present in large EVs isolated from damaged hearts. Knockout of *Neat 1* in mice led to impaired cardiac function, increased fibrosis, and increased fibrotic gene expression as compared to wild-type mice [171]. Hypoxia/reoxygenation of neonatal rat cardiomyocytes in vitro resulted in release of EVs that were enriched for miR-100-5p, -30d-5p, -21-5p, and -29b-3p [172].

TGF-β-treated rat cardiomyocytes in vitro released miR-21-enriched EVs that stimulated AKT-mediated fibrogenesis in target cardiac fibroblasts by inhibiting *PTEN* expression [173]. The cholesterol-lowering drug, simvastatin, protected against Ang II-induced cardiac fibrosis by blocking the interaction of EVs from Ang II-treated cardiomyocytes with target cardiac fibroblasts, by preventing EV-mediated differentiation of fibroblasts into myofibroblasts, and by modulating vesicular fibrosis-associated proteins that are required for fibroblast collagen production [174]. In addition, treatment of induced pluripotent stem cell-derived cardiomyocytes with sacubitril (neprilysin inhibitor) and valsartan (angiotensin receptor blocker) resulted in increased exosome production but decreased exosomal miR-181a levels [175]. In a chronic rat MI model, these drugs resulted in decreased miR-181a levels in plasma EVs and improved cardiac function, reduced fibrosis and hypertrophy, with these outcomes also being achieved by downregulation of miR-181a. Thus, miR-181 is a driver of cardiac dysfunction and fibrosis and its suppression in exosomes by sacubitril/valsartan underlies the pharmacological action of these drugs in cardiac repair [175].

#### 6.2.2. Production and Action of EVs by Fibroblasts

Elevated levels of miR-27a, -28-3p, and -34a, which are predicted to target nuclear factor erythroid 2-related factor 2 (*Nrf2*) were present in the infarcted heart and these miRs were enriched in TNF-α-stimulated EVs that mediated cross-talk between cultured cardiomyocytes and fibroblasts [176]. In the post-MI heart, EV delivery of *Nrf*-targeting miRs from fibroblasts to cardiomyocytes were proposed to disrupt *Nrf*-regulated antioxidant enzymes [176]. When added to cardiac fibroblasts, exosomes from Wnt3a-producing mouse fibroblast L cells activated canonical β-catenin-dependent signaling and exacerbated TGF-β-mediated fibrogenic signaling, while Wnt5a-enriched exosomes partially activated non-canonical Wnt pathways [177].

#### 6.2.3. Production and Action of EVs from T Cells

Infiltrating CD4+ T cells are important drivers of inflammation and fibrosis after MI [178] and exosomes from activated CD4+ T cells promoted cardiac fibroblast activation in vitro and exacerbated fibrosis and heart dysfunction in mouse MI [179]. This effect was attributed to exosomal 142-3p, which promoted Wnt signaling and cardiac fibroblast activation by attenuating expression of the Wnt suppressor molecule, adenomatous polyposis coli [179].

#### 6.2.4. Production and Action of EVs from Macrophages

Levels of the mRNA-stabilizing human antigen R (HuR) protein were increased in myocardium from diabetic ischemic human hearts and in cardiac or bone marrow-derived macrophages from *db/db* mice [180]. Exosomes from cultured macrophages exposed to high glucose induced expression of inflammatory or fibrogenic genes in fibroblasts in vitro but this effect was abrogated when EVs were rendered HuR-deficient. Moreover, cardiac fibrosis and left ventricle dysfunction in Ang II-induced diabetes in mice was attenuated by administration of HuR-deficient macrophage exosomes [180]. Also, miR-155 in macrophage EVs has shown to favor a profibrotic cardiac environment in several studies. For example, following MI in mice, miR-155 was upregulated in cardiac macrophages from which it was released in EVs that mediated its transfer into cardiac fibroblasts where it inhibited proliferation and promoted inflammation by suppressing expression of, respectively, *Son of Sevenless 1* and *SOCS1* [181] while, in a model of uremic cardiomyopathy in mice, miR-155 in macrophage EVs targeted *Foxo3a* in cardiomyocytes, resulting in pyroptosis (pro-inflammatory programmed cell death) and downstream cardiac hypertrophy and fibrosis [182].

#### 6.2.5. Production and Action of EVs from the Circulation

Serum exosomes from older rats were more effective than those from younger rats in promoting cardiac fibroblast proliferation and transdifferentation and this was correlated with an age-dependent decrease in exosomal HSP70 levels [183]. The proliferative and fibrogenic actions of serum exosomes could be ‘tuned” by experimentally manipulating the amount of exosomal HSP70 present [183].

**Figure 5:**
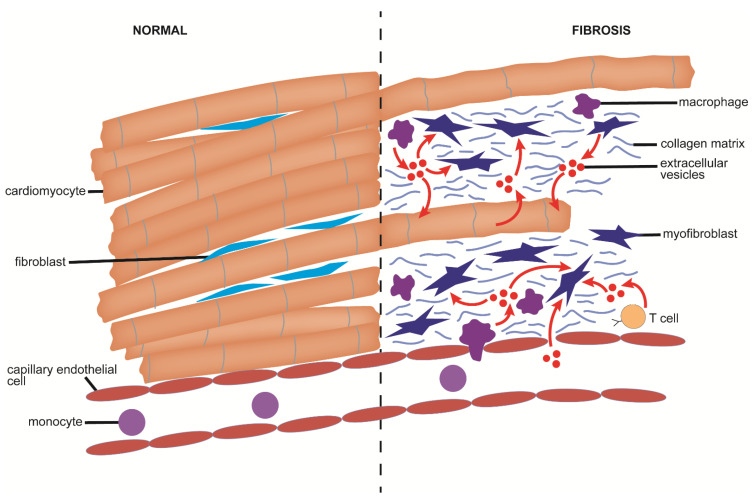
Proposed EV pathways in the pathogenesis of cardiac fibrosis. Cardiomyocytes exposed to stress such as hypoxia produce EVs that promote mesenchymal transition, survival, and fibrogenesis in cardiac fibroblasts/myofibroblasts in part due to delivery of enriched EV cargo components that include Neat 1, miR-29b-3p, miR-30d-5p, miR-100-5p, miR181a, miR-21 and miR-208a, the latter two of which target, respectively, AKT-*PTEN* and *Dyrk2*. TNF-α-stimulated cardiomyocytes and cardiac fibroblasts produce EVs that are exchanged between them and which can promote increased oxidative stress by delivery of *Nrf2*-tartgeting miR-27a, -28-3p, or -34a, while profibrotic pathways in cardiac fibroblasts are promoted by delivery of fibroblast EVs enriched with Wnt3a or Wnt5a. High glucose-stimulated macrophages produce EVs that are enriched in HuR which is required for EV-stimulated expression of inflammatory or fibrogenic genes in cardiac fibroblasts. Macrophage EVs are also enriched in miR-155 which exerts, firstly, anti-proliferative and pro-inflammatory effects in cardiac fibroblasts by targeting *Son of Sevenless 1/SOCS1* and, secondly, pyroptosis, hypertrophy and fibrosis in cardiomyocytes by its targeting of *Foxo3a*. Finally, age-related declines in HSP70 levels in serum EVs are associated with increased cardiac fibroblast proliferation. Only cells with a demonstrated role in EV production or response are shown; some of the depicted EV pathways are deduced from in vitro observations and have not been demonstrated in vivo. See text for details.

Some of the pathways of EV-regulated cardiac fibrosis discussed above are shown in Figure 5.

### 6.3. Therapeutic Actions of EVs in Cardiac Fibrosis

#### 6.3.1. EVs from Adult Stem Cells

EVs from human BM-MSC protected cardiomyocytes from oxidative stress-induced apoptosis stress, stimulated cardiomyocyte proliferation, and inhibited TGF-β-induced *αSMA* production in BJ fibroblasts [184]. These EVs improved cardiac function and increased angiogenesis, and decreased inflammation and fibrosis in rats MI models and were enriched in miRs that were predicted to target PI3-AKT and mTOR [184]. Administration of miR-101a-enriched BM-MSC EVs in post-MI mice resulted in improved heart function and anti-inflammatory and anti-fibrotic effects and were due to EV-mediated polarization of macrophages to the anti-inflammatory phenotype, dampening of *TGF-β* expression, and inhibition of autophagy [185]. BM-MSC exosomes that contained elevated levels of HIF-1α promoted in vitro endothelial cell angiogenesis, migration, proliferation and expression of angiogenic factors while preserving cardiac function, promoting angiogenesis, and reducing fibrosis in a rat MI model [186]. The therapeutic effect of EVs from human BM-MSC on endothelial function, myocyte survival, and fibrosis in rat MI models was reduced in older versus younger BM-MSC donors and attributed to the age-related loss of miR-221-3p which otherwise suppressed apoptosis through its inhibition of *PTEN* and stimulation of AKT activity [187]. BM-MSC exosomes engineered to contain the membrane Lamp2b fused to ischemic myocardium-targeting peptide were more efficiently internalized by hypoxic cardiomyocytes in vitro and showed enhanced accumulation in the post-MI heart in mice, resulting in improved effects in vivo including reduced myocardial inflammation, apoptosis, and fibrosis while improving vascularization and cardiac function [188]. Post-MI therapy by BM-MSC EVs was not improved using cell-free hydrogel formulations used to facilitate effectiveness of other cardiac therapies [189]. Aortic constriction in mice resulted in pressure overload-induced cardiac hypertrophy, myocardial apoptosis, and fibrosis that was attenuated by administration of mouse BM-MSC exosomes which, in vitro, prevented myocyte hypertrophy and apoptosis while promoting senescence of cardiac myofibroblasts [190].

Ischemia of BM-MSC also results in production of therapeutic EVs, which may be more potent than their normoxic counterparts. Thus, exposure of BM-MSC to ischemia resulted in the production of miR-22-enriched exosomes that reduced apoptosis in ischemic cardiomyocytes by miR-22 targeting of methyl CpG binding protein 2 [191]. These EVs reduced collagen deposition and infarct size in mouse MI models, and the anti-fibrotic effect was abrogated by inhibiting miR-22 production in the exosome donor BM-MSC [191]. EV therapy was enhanced by exposure of the donor BM-MSC to hypoxia, which resulted in elevated cellular and EV miR-210 and nSMase2 activity [192].

The therapeutic efficacy of cardiac stem cells in a post-MI model in rats was substantially improved by their pre-exposure to BM-MSC exosomes, as evidenced by improved cardiac stem cell survival, cardiac vascularization and function, and reduced fibrosis [193]. Exosome treatment resulted in modulation of multiple miRs that were predicted to impact genes involved in cardiac repair and regeneration [193].

Muscle satellite cells engineered to produce miR-26a-enriched muscle-targeted exosomes dampened fibrotic responses in the cardiac and skeletal muscle of CKD mice, reduced muscle wasting, and improved cardiac function [194].

In vitro, EVs from UMSC increased the Bcl2: Bax ratio in hypoxic myocardial cells and stimulated endothelial cell migration and tube formation [195]. In acute MI in rats, UMSC EVs improved lung function, reduced fibrosis and apoptosis, and stimulated cardiomyocyte proliferation [195] and in vivo therapeutic effects were enhanced by encapsulation of the EVs in cardioprotective peptide hydrogel [196]. The therapeutic effect of UMSC EVs also involved targeting of the *Bim* apoptosis gene by exosomal miR-24 while immune rejection of BM-MSC used for cell-based therapy was prevented and cardiac repair was enhanced by knockout of human leucocyte antigen light chain β2 microglobulin to modulate exosome imprinting [184]. In a mouse MI model, the therapeutic properties of BM-MSC from elderly human donors was improved by pretreating the cells with EVs from UMSC, which resulted in the loss of senescence and apoptosis from the BM-MSC and their prolonged survival in vivo resulting in improved cardiac function, increased cardiac vessel density, and decreased scar [197]. This improved outcome was attributed to the effect of EV miR-136, which inhibited expression of apoptotic peptidase activating factor [197]. In type 2 diabetes mellitus stroke mice, administration of exosomes from human umbilical cord blood derived stem cells improved cardiac function, increased myocardial vascularization, and decreased cardiomyocyte hypertrophy, oxidative stress, interstitial fibrosis and expression of *TGF-β* [198]. These exosomes were enriched in miR-126 and caused cardiac expression of its targets (*MCP1*, *VCAM1*, *Spred-1*) to be attenuated [198].

EVs from AD-MSCs were therapeutic for post-MI heart damage by attenuating inflammation, apoptosis, and fibrosis and promoting macrophage M2 polarization and angiogenesis [199,200,201]. EVs targeted the S1P/SK1/S1PR1 axis [199] and their therapeutic effects were augmented with either miR-126 to target angiogenic and cardiac repair pathways [200] or miR-146a to targeted early growth response factor 1 [201]. In a pig model of co-exisiting metabolic syndrome and renal stenosis, intra-renal administration of AD-MSC EVs resulted in attenuation of cardiac injury and dysfunction, including improved myocyte and capillary density and reduced inflammatory cytokine expression and fibrosis [202].

Cardiac mesenchymal stem cells CMSC are a subpopulation of predominantly Sca-1+ MSC that arise from cardiac tissue and play a role in cardiac regeneration. Exposure of ischemic Sca-1+ CMSC to heat shock resulted in enhanced cell survival due to regulation of a heat shock factor 1/miR-34a/HSP70 axis [203]. CMSC exosomes promoted the same pro-survival pathway in oxygen/glucose deprived cardiomyocytes suggesting that they contribute to CMSC-mediated reduced cell death and fibrosis in ischemic heart in vivo [203]. EVs from *Notch*-overexpressing CMSC were therapeutic in post-MI mouse heart, resulting in neovascularization of the infarct area, decreased cardiac fibrosis, and improved cardiac function [204].

EVs from cardiac-resident mesenchymal progenitor cells (CPC) blocked oxidative stress in cardiomyocytes exposed to doxorubicin and trastuzumab, which have off-target cardiac toxicity when used clinically to treat breast cancer [205]. CPC EVs were therapeutic in a rat model of doxorubicin/trastuzumab-induced myocardial fibrosis, inflammation, inducible nitric oxide synthase (iNOS), and left ventricular dysfunction and this was due to suppression of genes involved in inflammation, innate immunity, and death by EV miR-146a-5p [205]. The therapeutic actions of subcutaneously implanted CPCs in rat MI involved transit to the hearts of CPC EVs carrying cardiac repair proteins [206].

Treatment in vitro of dermal fibroblasts with EVs from rat cardiosphere cells (CDCs), which are obtained from CPCs via cardiosphere formation resulted in a phenotypic and functional reprogramming whereby “therapeutic” apoptotic and angiogenic cells were produced that contained cardioprotective miRs typical of the EV producer CDC [207]. Moreover, these EV-primed fibroblasts, or the EVs themselves, were therapeutic in rat chronic MI, resulting in improved left ventricular ejection fraction, reduced scarring, and increased microvessel density [207]. Similar results were obtained after administration of human CDC EVs in porcine models of acute or chronic MI [208] or pediatric dilated cardiomyopathy [209]. *Wnt/β-catenin* signaling in the CDC was critical for the potency of CDC and proposed to enhance an antifibrotic EV miR-92-*BMP* pathway [210]. Overexpression of *β-catenin* and *Gata4* in fibroblasts imparted therapeutic properties on the cells or their EVs as shown by, respectively, improved survival and heart function in mouse MI models or functional improvement and anti-fibrotic effects in the skeletal muscle of the *mdx* mouse model of Duchenne muscular dystrophy [210]. In the dilated cardiomyopathy model, the improvement in cardiac function and reduced fibrosis was attributed to the enrichment in CDC EVs of proangiogenic and cardioprotective miRs including miR-146a-5p which attenuated fibrosis by reducing inflammation and apoptosis through its targeting of TRAF6/Smad4/FOS [209]. CPC-derived exosomes also resulted in improvement of heart function and reduced apoptosis and fibrosis when administered to a mouse model of doxorubicin-induced dilated cardiomyopathy [211] and the therapeutic outcome was improved by conjugation of the exosomes with cardiac homing peptide which targets ischemic myocardium [212]. Human trophoblast-derived exosomes had similar therapeutic outcomes in the same injury model, with the effects attributed to EV-mediated suppression of a mir-200b-*Zeb1* axis in cardiomyocytes [213].

Endothelial progenitor cells that were exposed to hypoxia/reoxygenation produced exosomes that promoted mesenchymal to endothelial transition in cultured cardiac fibroblasts [214]. This pro-angiogenic and anti-fibrotic activity was attributed to the action of exosomal miR-133 [215]. Exosomes from endothelial colony-forming cells (ECFCs) attenuated activation of cardiac fibroblasts in vitro when the EV donor ECFC were grown under normoxic but not hypoxic conditions [216]. The therapeutic effect was attributed to exosomal miR-10b-5p, which was suppressed during hypoxia but which, in normoxic conditions, attenuated expression of the fibrosis-related genes *Smurd 1* and *HDAC4* [216].

Telocytes are interstitial cells that occur in multiple organs and which, in the heart, play a supportive role in the stem cell niche and function in post-MI regeneration. Exosomes from cardiac telocytes promoted a pro-angiogenic phenotype when added to cultured endothelial cells and caused improved cardiac function and angiogenesis and decreased fibrosis when administered in a rat MI model [217]. Delivery of exosomes from myogenic progenitor cells into the hearts of *MDX* Duchenne muscular dystrophy mice caused cardiac levels of dystrophin to be restored and improved myocardial function [218].

#### 6.3.2. EVs from Induced Pluripotent Stem Cells (iPSC)

Exosomes from iPSC-derived cardiomyocytes (iCM) stimulated cardiomyocyte survival under hypoxic conditions and enhanced cardiac function and decreased apoptosis and fibrosis in mouse MI [219]. iCM exosomes targeted mTOR signaling and autophagy and elicited therapeutic and transcriptomic changes in the ischemic heart that were comparable to iCM themselves [219]. CPCs induced from human iPSCs produced EVs that inhibited TGF-β-induced fibrotic gene expression in cultured fibroblasts in vitro and attenuated cardiac dysfunction and fibrosis in mouse MI in vivo [220]. The EVs were enriched in miRs associated with promoting angiogenesis and cardiomyocyte proliferation or inhibiting fibrosis, the latter involving targeting of growth differentiation factor-11 (*GDF-11*) or *ROCK-2* by miR-373 [220].

#### 6.3.3. EVs from Embryonic stem Cells (ESC)

In MI models, myocardial administration of ESC exosomes enhanced left ventricular function, neovascularization, cardiomyocyte survival, expansion of CPC and reduced fibrosis [221]. Moreover, in vitro pre-treatment of CPCs with ESC exosomes resulted in augmentation of the ability of CPCs themselves to improve cardiac function and reduce fibrosis post-MI and this was attributed to the effect of exosomal miR-294, which promoted CPC survival, cell cycle progression, and proliferation [221]. In mouse MI models, EVs from cardiac-committed ESCs were as effective as the cells themselves in improving heart function, decreasing infarct size and modulating cardiac gene expression [222]. On the other hand, whereas in a porcine MI model intravenous injection of EVs from the *E1-MYC 16.3* ESC-derived MSC line resulted in a 30% decreased infarct size and somewhat improved regional and global LV functions, fibrosis was accelerated rather than diminished and likely accounted for sub-optimal EV therapeutic effects on heart function [223].

#### 6.3.4. EVs from Cardiomyocytes or Skeletal Muscle

In diabetic mice, levels of cardiomyocyte exosomal miR-29b and -455 were increased during exercise and were associated with exercise-mediated improvements in cardiac remodeling through their ability to inhibit MMP9 activity [224]. MiR-378 in cardiomyocyte EVs was implicated in inhibiting fibrogenic gene expression in cardiac fibroblasts by targeting mitogen-activated protein kinase kinase 6 and downstream p38 MAPK signaling, consistent with a role for miR-378 in cardiac dysfunction, hypertrophy, and fibrosis in a mouse pressure overload model [225]. In rat MI, remote ischemic conditioning by repeated bilateral hindlimb ischemia/reperfusion resulted in improvement of cardiac function, reduced interstitial fibrosis and reduced oxidant stress and this outcome was suggested to involve systemic delivery from skeletal muscle to cardiomyocytes of exosomes enriched in anti-fibrotic miR-29a and cardioprotective insulin-like growth factor-1 receptor (IGF-1R) [226].

#### 6.3.5. EVs from Vascular Endothelial or Smooth Muscle Cells

Increased numbers of exosomes were released from cultured human umbilical vein endothelial cells exposed to shock waves and these EVs were angiogenic due in part to the presence of pro-angiogenic miR-19a-3p. When administered in vivo to a mouse MI model, these EVs preserved ventricular function and angiogenesis and decreased the degree of fibrosis in the infarct area [227]. In a study of vascular inflammatory mediators, EVs from LPS-treated endothelial cells stimulated endothelial dysfunction as shown by increased production of ICAM protein, ROS, and onset of cellular senescence but decreased levels of phospho-endothelial nitric oxide synthase and pAKT, while EVs from vascular smooth muscle cells produced increased levels of anti-fibrotic MMP-9 [228].

#### 6.3.6. EVs from the Circulation

Hypertension is a progressive chronic disease that can lead to coronary artery disease, stroke and CKD, with fibrotic manifestations in the heart and kidney. Plasma exosomes from normotensive rats caused decreased blood pressure and prevented perivascular cardiac fibrosis when transferred into hypertensive rats and, conversely, plasma exosomes from hypertensive rats caused increased blood pressure and increased perivascular fibrosis when transferred into normotensive rats [229]. In another study, administration of miR-21-loaded human blood exosomes to hypoxic cardiomyocytes in vitro resulted in down-regulation of *Smad7* and *PTEN* and upregulation of *MMP2* while cardiac dysfunction and fibrosis in mouse MI were exacerbated or blocked by exosomes enriched with, respectively, miR-21 or miR-21 inhibitor [230].

### 6.4. EVs as Biomarkers in Cardiac Fibrosis

Exosomes from the serum of patients with persistent atrial fibrillation contained levels of miRs -103a, -107, -320d, -486, and let-7b that were elevated at least 4.5-fold as compared to control patients [231]. These miRNAs targeted pathways related to atrial function and structure, oxidative stress, and fibrosis [231]. Similarly, EVs from pericardial fluid of atrial fibrillation patients contained 31 downregulated miRs and 24 upregulated miRs, the latter of which included miR-382-3p, -2136-5p, and 450a-2-3p which are predicted to target multiple fibrosis-related genes [232]. Plasma exosomes from heart failure patients contained elevated levels of miR-21 and reduced levels of miR-425 and miR-744 and these miRs regulated fibrogenic pathways in cardiomyocytes [173,233].

## 7. Hepatic Fibrosis

### 7.1. Causes and Pathological Features of Hepatic Fibrosis

Chronic liver injuries affect millions of people world-wide and are characterized by prolonged hepatic injury, inflammation, and fibrosis, the latter increasing the risk of life-threatening complications such as cirrhosis, portal hypertension, hepatocarcinoma, and organ failure [234]. The principal fibrosis-producing cells of the liver are hepatic stellate cells (HSC) which undergo activation and play a transient self-limiting role in would repair in acute injury but which unrelentingly produce large amounts of collagenous scar during chronic liver injury [235]. Portal fibroblasts in the periportal mesenchyme surrounding the bile ducts may also produce fibrotic scar, especially after cholestatic liver injury [236].

Alcoholic liver disease (ALD) describes a spectrum of hepatic pathologies that are caused by chronic alcohol consumption [237]. Hepatocytes are the principal site of ethanol metabolism but the action of alcohol dehydrogenase or cytochrome P450 2E1 (CYP2E1) yields highly toxic acetaldehyde as an intermediate metabolite. Alcohol exerts additional direct toxic effects by its stimulation first, of ROS via CYP2E1-mediated mitochondrial influx of reduced NADPH or TNF-α mediated interaction of *N*- acetyl-sphingosine with mitochondria and second, of reactive nitrogen species by inducible nitric oxide synthase. Chronic alcohol consumption also induces alcoholic steatosis due to inhibition of fatty acid oxidation, upregulation of lipogenic genes, and altered lipid transport. In some cases, the disease advances to inflammation (alcoholic steatohepatitis) in which infiltration of macrophages and neutrophils is driven by gut-derived pathogen-associated molecular patterns (e.g., LPS), damage associated molecular patterns from dying hepatocytes, and inflammatory cytokines from hepatocytes or Kupffer cells (KC). With continued alcohol consumption, HSC may become activated by the combined effects of alcohol, acetaldehyde, ROS, KC activity, and inhibition of intrinsic anti-fibrotic pathways, resulting in progression to fibrosis and cirrhosis in some individuals [237]. Likewise, non-alcoholic fatty liver disease (NAFLD) encompasses a spectrum of diseases of increasing severity from simple fatty liver (steatosis) to non-alcoholic steatohepatitis (NASH) and cirrhosis [238]. About 20% of NAFLD patients progress to NASH, a subset of whom will progress to fibrosis and cirrhosis [239]. NASH is initiated by multiple hits to the liver (insulin resistance, obesity, diabetes, gut endotoxin, hyperlipidemia) causing lipotoxicity-induced oxidative or endoplasmic reticulum stress in hepatocytes, which triggers similar pathogenic pathways as in ALD [240,241].

Chronic infection with hepatitis B or C virus (HBV, HBC) results in severe parenchymal injury leading to fibrosis, cirrhosis, and hepatocarcinoma. The use of sofosbuvir (which inhibits HCV NS5B protein) as a first line treatment for all HCV genotypes has virtually eradicated HCV while the HBV vaccine (which targets HBsAg viral envelope protein) is highly efficacious. However, not all individuals are effectively treated (due to drug cost, accessibility, non-compliance, etc.) and even when the primary disease is successfully treated, hepatic fibrosis may still persist. Hepatic fibrosis, cirrhosis, and hepatocarcinoma thus remain significant complications of HBV or HCV infection for millions of people worldwide.

The role of EVs in hepatic fibrosis has been determined using several in vivo liver injury models, including administration of chemicals, consumption of special diets to cause NASH-like pathology, surgery, or parasitic infections [242].

### 7.2. Mechanistic Aspects of EVs in Hepatic Fibrosis

#### 7.2.1. Production and Action of EVs from Hepatocytes

Hepatocytes produce increased numbers of EVs when cultured in the presence of free fatty acids such as palmitic acid to mimic the lipotoxicity seen in NAFLD and this is due activation of ROCK-1 via a C/EBP homologous protein/DR5/caspase8/caspase 3 proapoptotic axis [243,244,245]. EVs from lipotoxic hepatocytes stimulated migration, proliferation, activation, and fibrogenesis in human or mouse HSC in vitro [245,246]. HSC activation was dependent on the presence of vannin on the EV surface, while fibrogenesis was stimulated by EV miR-128-3p, -27b, or -130b, which targeted *PPARγ* [246]. EVs from lipotoxic hepatocytes contained enhanced levels of NASH-related miRs including miR-24, -19b, -34a, -122, and -192, the latter two of which were also enhanced in serum exosomes from patients with advanced NAFLD (S2-3 steatosis, F2-F4 fibrosis) [245]. These EVs, or those from the serum of experimental NASH in mice or of human NASH patients, induced expression and release of proinflammatory cytokines (IL-1β, IL-6) in macrophages and this was dependent on the presence of TNF-related apoptosis-inducing ligand, a DR5 ligand, in the EV payload [244]. Internalization of EVs from fat-laden hepatocytes caused NF-κB-mediated activation of NLRP3 inflammasome in hepatocytes and macrophages resulting in increased IL-1β release [247].

Mice fed choline-deficient L-amino acid defined diet or high fat diet had increased numbers of circulating EVs that were correlated with progression of NAFLD [248]. Some circulating EVs in NAFLD animals were likely of hepatocyte origin because they contained liver carboxylesterase, asialoglycoprotein-receptor, mir-122 and miR-192 which are principally hepatocyte-associated [248]. In mice fed trans-fatty acids, fructose, and cholesterol (HTF-C) diet, hepatic fibrosis and expression of HSC markers was blocked and reversed by the thiazolidinedione, MSDC-0602 [249]. When added to HSC in vitro, plasma EVs from HTF-C-fed mice stimulated expression of fibrotic markers whereas those from mice receiving MSCD-0602 or that were null for mitochondrial pyruvate carrier 2 (*MPC2;* a thiazolidinedione-binding moiety) did not. These data show that plasma EVs, possibly of hepatocyte origin, stimulate HSC activation in NASH/NAFLD [249]. In HTF-C-fed mice, the frequency of circulating hepatocyte-derived EVs was increased and this was reduced using fausidil, a ROCK inhibitor which also reduced serum alanine transaminase (ALT), hepatic expression of *mac1*, *IL-1β*, and *αSMA*, and collagen deposition [244]. EVs from lipotoxic hepatocytes were enriched with activated integrin β1 and enhanced integrin β1-mediated monocyte adhesion to liver sinusoidal endothelial cells under conditions of shear stress [243]. Lipotoxic hepatocytes released increased numbers of EVs into the circulation of NASH mice and, further, serum EVs from NASH patients with Stage 1-2 hepatic fibrosis contained significantly more integrin β1 than EVs from patients with simple steatosis. In mouse NASH, proinflammatory monocyte infiltration, hepatocyte apoptosis and injury, and expression of fibrotic markers collagen 1α1 and osteopontin were decreased by anti-integrin β1 antibody supporting a role for circulating hepatocyte EV integrin-β1 in these processes [243].

As compared to EVs from control hepatocytes, those produced by hepatocytes isolated from a mouse model of alcoholic hepatitis were increased 10-fold in frequency and stimulated *αSMA* and *collagen 1α1* expression in HSC or Toll-like receptor 9 (TLR9)-dependent IL-1β and IL-17 production in hepatic macrophages [250]. The EVs contained firstly, 4 downregulated and 20 upregulated miRs, the latter including miR-27a and miR-181 which were predicted to target *Smad7* and the *Nrld2* quiescence marker and secondly, a 10-fold enrichment of organelle proteins and mitochondrial DNA. This elegant study suggested that in the context of alcoholic hepatitis, hepatocyte EVs drive fibrosis by direct miR-mediated regulation in HSC and by production of profibrogenic cytokines by macrophages [250]. In mouse models of acute liver injury and early fibrosis, hepatocyte exosomes increased *TLR3* expression in HSC [251]. This resulted in HSC activation and increased their production of IL-17A, IL-1β, and IL-23 which acted locally to amplify ll-17A production in nearby δT cells. Self-coding TLR3 ligands and exosomes produced by damaged hepatocytes were propose to act in concert to drive expression and activation of TLR3 in HSC, resulting in a positive feedback mechanism between HSC and δT cells that drives 1l-17A production and exacerbates hepatic fibrosis [251]. In hepatocytes, the stress protein tribbles pseudokinase 3 (TRIB3) mediated profibrotic effects by suppressing autophagic degradation of late endosomes and increasing the production of EVs that are enriched in inhibin beta A/activin A and which stimulated migration, proliferation and activation of HSCs [252]. Metastasis-associated lung adenocarcinoma transcript 1 (*MALAT1*), a lncRNA that is upregulated in hepatic fibrosis and activated HSC, was present in exosomes released by L-02 human fetal hepatocytes exposed to fibrosis-causing arsenite [253]. Incubation of these EVs with cultured human LX2 HSCs resulted in the delivery of MALAT1 to the cells which contributed to their activation by suppressing miRNA-26b, a negative regulator of *collagen 1α2* [253]. Exposure of rats to high concentrations of heavy metals resulted in liver injury that included hepatocyte damage, inflammation, and fibrosis and this was accompanied by an increased frequency of EV shedding [254].

Exosomes from HCV-infected hepatocytes were enriched in miR-19a, -20a, and -195 and stimulated expression of profibrotic markers in HSC [255]. MiR-19a was correlated with advanced hepatic fibrosis in serum exosomes from HCV patients and, in HSC, directly stimulated fibrotic gene expression and inhibited *SOCS3* expression via downstream activation of the STAT3/TGF-β1/Smad3 axis, the latter also occurring in response to exosomes from HCV-infected hepatocytes [255]. HCV-replicating human hepatocytes delivered EV miR-192 into co-cultured human HSC, HSC resulting in upregulation of its *TGF-β* target and downstream stimulation of fibrogenic gene expression and transdifferentiation [256].

#### 7.2.2. Production and Action of EVs from Cholangiocytes

Cholangiopathies such as primary sclerosing cholangitis involve epithelial damage and inflammation in the bile duct, resulting in blockage of the duct and fibrosis. Cultured mouse large cholangiocytes (MLE) produced more EVs when they were treated with cholestatic estrogen or taurocholate acid and these EVs contained increased levels of *H19*, a lncRNA that is preferentially expressed in cholangiocytes and associated with cholestatic injury [257]. In mouse fibrotic or cholangiopathic models, delivery of exosomal *H19* from cholangiocytes to hepatocytes, macrophages, or HSC drove cholestatic injury, inflammation, and fibrosis [257,258,259]. In addition, plasma EVs from the *Mdr2^−/−^* primary sclerosing cholangitis mouse model stimulated macrophage activation in vitro and localized to hepatic monocytes in vivo, consistent with the identification of an EGFR-mediated pathway of macrophage activation triggered by EVs from senescent cholangiocytes [260].

#### 7.2.3. Production and Action of EVs from HSC or Fibroblasts

Treatment of human HSC in vitro with platelet-derived growth factor (PDGF) resulted in a >50% increase in EV release and a >2-fold increase in the concentration of 440 EV proteins [261]. One such protein was PDGF receptor alpha (PDGFRα) and its enrichment in EVs was dependent on Src homology region 2 domain-containing phosphatase-2 (SHP2) which binds to Tyr720, and resulted in the ability of the EVs to stimulate HSC migration in vitro and collagen deposition and/or fibrotic gene expression under basal conditions or in carbon tetrachloride (CCl_4_) or bile duct ligation (BDL) hepatic fibrosis models. PDGFRα levels were enhanced in serum EVs from ALD patients with hepatic fibrosis or mice with CCl_4_ or BDL hepatic fibrosis [261]. The release of pro-fibrotic EVs from activated HSC by PDGF or SHP2 occurred through the activation of the mTOR pathway, which caused increased exosome release via inhibition of autophagy and increased microvesicle release via activation of ROCK [262]. Exosome release and autophagy were also mechanistically linked by decreased levels of miR-30a in exosomes from activated HSC which was consistent with the downregulation of miR-30a in mouse models of hepatic fibrosis and its ability to suppresses HSC autophagy, activation, and fibrogenesis by attenuating of beclin1 expression [263]. Additionally, exosome release from activated HSC was *HIF-1*-dependent and the resultant exosomes contained *HIF-1*-dependent glucose transporter 1 (GLUT1) and pyruvate kinase M2 (PKM2) which were subsequently delivered to quiescent HSC, KCs, or luminal sinusoidal endothelial cells (LSECs) [264]. Furthermore, profibrotic CCN2 in activated HSC was packaged into their secreted EVs which, upon binding to target HSC via cell surface integrins and heparan sulfate proteoglycans, caused elevated intracellular *CCN2* and *αSMA* levels [36,265], while stimulation by Ang II of HSC fibrogenesis and endoplasmic reticulum stress in vitro was mediated by apoptosis signaling regulating kinase 1 and resulted in the liberation of pro-fibrogenic EVs [266].

Autonomous HSC activation in vitro was associated with a 4.5-fold increase in EV production and a switch in EV bioactivity such that EVs from quiescent HSC suppressed HSC fibrogenic gene expression whereas EVs from activated HSC stimulated HSC fibrogenesis and activation [267]. This switch was accompanied by a dramatic increase in EV proteomic complexity in which the number of EV proteins increased from 46 proteins during quiescence (with histones and keratins predominating) to 337 proteins after activation (with the principal proteins associated with extracellular spaces or ECM, proteasome, collagens, vesicular transport, metabolic enzymes, ribosomes and chaperones) [267].

EVs from portal myofibroblasts were proposed to function in hepatic vascular remodeling and the production of scar tissue because progression to cirrhosis involved the production of collagen 15A1-positive portal myofibroblasts, which localized to vessels in fibrotic septa and released vascular endothelial growth factor A (VEGFA)-rich microparticles that stimulate VEGF receptor 2 activation and tube formation in endothelial cells [268].

#### 7.2.4. Production and Action of EVs from Endothelial Cells or Macrophages

EVs from immortalized liver sinusoidal endothelial cells activated AKT and migration in human LX2 HSC [269]. These effects were mediated by exosomal sphingosine kinase 1 (SK1) and sphingosine 1-phosphate (S1P), as well as exosomal FN which engaged integrin α5β1 on the HSC cell surface. Serum exosomes from human liver cirrhosis or alcoholic hepatitis or from mouse CCl_4_ or BDL hepatic fibrosis contained elevated SK1 or S1P [269]. Proliferation and activation of human LX2 HSC was stimulated by exosomes from LPS-primed THP-1 human monocytic leukemia macrophages to [270]. LPS treatment affected the concentrations of multiple exosomal miRs, including miR-103-3p, the levels of which were increased and caused suppression of its *KLF4* target, thus enhancing expression of *αSMA* and *collagen 1α1* [270].

#### 7.2.5. Action of EVs from the Circulation

Differential cytokine and miR expression was reported in plasma EVs from patients with alpha-1 antitrypsin deficiency (in whom hepatic inflammation and fibrosis occur due to accumulated misfolded ZAAT protein in hepatocytes) and these EVs activated JAK/STAT, NF-κB, and CXCR3/CXCL10 in human HSC in vitro [271]. In systemic mastocytosis (SM), a condition in which mast cells accumulate in excessive numbers in multiple organs and can result in hepatic fibrosis, serum EVs were enhanced and correlated with disease progression, contained common mast cell proteins and stimulated proliferation, fibrogenesis and activation in human LX2 HSC in vitro [272]. This interaction involved the delivery of phospho-KIT from EVs to HSC and its neutralization using blocking KIT antibody negated EV-mediated HSC proliferation and activation. Moreover, administration of SM-EVs in mice resulted in HSC activation and the delivery of EV-derived KIT into the HSC population [272].

**Figure 6:**
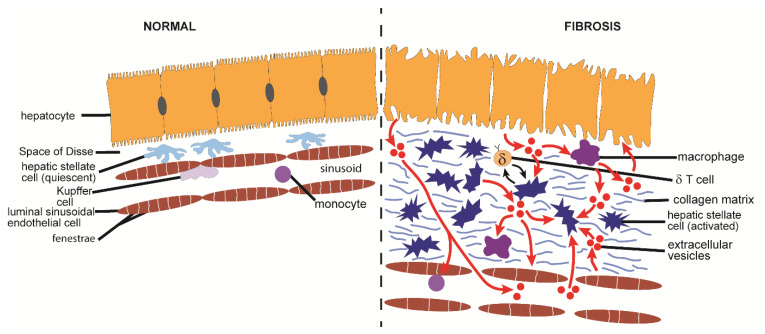
Proposed EV pathways in the pathogenesis of hepatic fibrosis. Hepatocytes that are injured by exposure to hepatitis viruses, alcohol, or free fatty acids produce increased numbers of EVs due to activation of EV biogenic components (e.g., ROCK-1) and suppression of autophagy-associated late endosomes. These EVs drive activation and function in macrophages or HSC, or alternatively are released into the circulation. Production by macrophages of pro-inflammatory cytokines (e.g., IL-1β, IL-6) occurs upon interactions of hepatocyte EVs with TLR and DR5 as well as stimulation of NF-κB-activated NLRP3 inflammasomes. Hepatocyte EVs also stimulate TLR3 expression in HSC which causes HSC activation and drives an IL-17A positive feedback loop between HSC and δ T cells that exacerbates inflammation and fibrosis. HSC activation and fibrogenesis is directly stimulated by hepatocyte EVs and involves various mechanisms such regulation of *PPARγ* by EV miR-128-3p, -27b, -130b, *Smad 7* by EV miR-27a, *Nrld2* by miR-181, *SOCS3* by EV miR-19a, *TGF-β* by miR-192, and miR-26b and its *collagen 1α2* target by EV MALAT. HSC themselves release EVs that are enriched in PDGFα, bind to HSC integrins and heparan sulfate proteoglycans, and stimulate HSC migration, activation, and fibrogenesis. EVs produced downstream of activation of apoptosis signaling regulating kinase 1 or HIF-1 during HSC activation deliver GLUT1 and PKM2 to quiescent HSC, KC or LSECs and are pro-fibrogenic in part due to suppressed EV miR-30a levels and a proteomic cargo that is enriched for ECM-, proteasome- and collagen-associated components. AKT activation and migration in HSC is stimulated by EVs from liver sinusoidal endothelial cells while vascular endothelial cells undergo tube formation in response to VEGF-enriched EVs from fibrotic myofibroblasts and demonstrate enhanced adhesion to monocytes in the presence of integrin- β1-enriched EVs from lipotoxic hepatocytes. Activated macrophages produce miR-103-3p-eriched EVs that stimulate HSC activation and fibrogenesis via suppression of *KLF4*. EVs in serum or plasma from fibrotic patients activate HSC and are likely derived from injured hepatocytes (e.g., NAFLD, alpha-1 antitrypsin deficiency) or mast cells (e.g., systemic mastocytosis). Only cells with a demonstrated role in EV production or response are shown; some of the depicted EV pathways are deduced from in vitro observations and have not been demonstrated in vivo. See text for details.

Some of the pathways of EV-regulated hepatic fibrosis discussed above are shown in Figure 6.

### 7.3. Therapeutic Actions of EVs in Hepatic Fibrosis

#### 7.3.1. EVs from Adult Stem Cells

In rats, administration of human BM-MSC EVs reduced CCl_4_-induced liver injury, fibrosis, oxidative stress, and circulating inflammatory cytokines while increasing hepatocyte proliferation [273]. Mechanistically, the therapeutic effect was attributed to exosome-mediated activation of *Wnt/β-catenin* as shown by reduced hepatic production of *PPARγ*, Wnt3a, *Wnt10b*, *β-catenin*, *WISP1*, *cyclin D1*, *αSMA*, and *collagen I* [273]. In studies relating to BM-MSC-augmented post-liver transplant regeneration, LPS-stimulated macrophages produced enhanced levels of fibrolytic MMPs when the cells were co-cultured with BM-MSC; this was associated with the action of miR-6769b-5p, which inhibited *ATF4* expression and was the most enriched miR in EVs from the co-culture supernatant, highlighting potential anti-fibrotic actions of EV miR-6769-5p [274].

EVs from AD-MSC engineered to over-produce miR-122 (a known inhibitor of fibrosis) suppressed proliferation and collagen production in activated human or mouse HSC with suppressed expression of *P4HA1*, *IGF-1R* and *CCNG1* target genes [275]. Administration of miR-122-enriched AD-MSC EVs in CCl_4_-treated mice resulted in attenuation of hepatic fibrosis, suppression of hepatic *TGF-β1* and *αSMA* expression, and inhibition of serum ALT, hyaluronan, type III procollagen, aspartate transaminase and liver hydroxyproline [275] while mir-150-5p-enriched AD-MSC EVs were anti-fibrotic due to targeting of *CXCL1* [276]. Similarly, EVs from miR-181-5p-over-expressing AD-MSCs were antifibrogenic in vitro and anti-fibrotic in vivo at least in part due to down regulation of *STAT3* and *Bcl2*, stimulation of autophagy, and suppression of fibrogenic gene expression [277]. CCl_4_-induced liver fibrosis in mice resulted in downregulated levels of hepatic *mmu_circ_0000623* and when mice were treated with EVs from AD-MSCs enriched in this circular RNA, liver fibrosis was suppressed due to promotion of miR-125/ATG4D-mediated autophagy of damaged cells, including hepatocytes [278].

In CCl_4_ mouse models, EVs from human UMSC caused a decrease in inflammation, acute liver injury, hepatic fibrosis, hepatic fibrotic gene expression, and hepatic tumor development [279,280]. These effects were accompanied by EV-mediated reduction in *TGF-β1/Smad* signaling and myofibroblast frequency [280] as well as reduced apoptosis and oxidant stress [279]. The EVs elicited similar effects when added to TGF-β1-treated HL7702 [280] or CCl_4_-treated L-02 hepatic cells in vitro [279]. EVs from human tonsil-derived mesenchymal stromal cells reduced expression of *αSMA*, *TGF-β*, *CCN2* and *vimentin* in human LX2 HSC in vitro and attenuated collagen deposition and profibrotic gene expression in CCl_4_-induced liver fibrosis in mice, with the therapeutic effect attributed to elevated levels of EV miR-486-5p which suppressed *Smo Hh* receptor expression and signaling in HSC [281]. EVs from human umbilical cord perivascular cells were antifibrotic and inhibited fibrosis-related gene expression in TAA-induced hepatic fibrosis in vivo but these effects were augmented upon prior loading of the EVs with IGF-1 by adenoviral transduction of the donor cells [282]. These EVs were also inhibitory for fibrogenesis in HSC in vitro as well as modulating inflammatory mediators (iNOS, arginase, IL-6, TNF-α) in cultured macrophages [282].

In CCl_4_-treated mice or rats, hepatic fibrosis was suppressed by administration of EVs from human AECs [283,284]. The therapeutic effect in vivo was linked to the presence in EVs of MFGE8, HSP72, and superoxide dismutase 1 (SOD1) which attenuate TGF-β-regulated processes such as fibrosis and myofibroblast production as well as decreased macrophage infiltration and with increased macrophage polarization to the M2 phenotype, possibly by regulation of PI3K/Ak [283,284]. Similar outcomes occurred in rat high fat diet NASH models in which hepatic inflammation, inflammatory cytokines and KC activation were reduced by AEC EVs [284].

Administration of EVs from human liver stem cells to SCID mice with diet-induced NASH resulted in decreased serum ALT, hepatic fibrosis and expression of fibrosis- and inflammation-related genes [285]. CCl_4_-induced fibrosis in rats was also reduced by liver stem cell EVs and this effect was enhanced by co-administration with the tyrosine kinase inhibitor Nilotinib [286]. Liver stem cell-derived EVs reduced the ductular reaction, cholangiocyte growth, hepatic fibrosis, and HSC activation in *Mrd2^−/−^* mice and this was attributed to the action of let-7 in the EVs, which caused multiple targets of let-7 to be suppressed in vivo or in cultured cholangiocytes [287]. HSC were deactivated in culture when treated with supernatant from EV-treated cholangiocytes suggesting that liver stem cell EV therapy of cholangiopathic injuries is likely attributable to their direct effects on cholangiocytes and indirect effects on HSC [287].

#### 7.3.2. EVs from iPSCs

Human iPSCs obtained by reprogramming primary skin fibroblasts released EVs that contained high levels of 22 miRs including miR-92a-3p, 302-3p, and 10b-5p which have potential antifibrotic properties [288]. In cultured TGF-β-treated human primary HSC, iPSC EVs suppressed fibrogenic gene expression, chemotaxis, and proliferation and resulted in significant up- or down-regulation of 295 genes. iPSC EVs suppressed hepatic fibrosis in mouse CCl_4_ and BDL injury models [288].

#### 7.3.3. EVs from ESC

Administration of EVs from human ESC during thioacetamide (TAA) administration in rats resulted in less chronic liver injury as shown by reduced hepatic fibrosis, suppressed expression of fibrogenic, inflammatory and apoptotic genes, and increased expression of collagenases, anti-apoptotic genes, and anti-inflammatory molecules [289].

#### 7.3.4. EVs from Hepatocytes

EVs produced by mouse hepatocytes bound to integrins αvβ3 and α5β1 on the surface of activated mouse HSC or ethanol-treated mouse hepatocytes in which, respectively, fibrogenesis and cell death were subsequently suppressed [265,290,291]. Similarly, human hepatocytes produced EVs that dampened activation and fibrogenesis in activated mouse HSC and which rescued human or mouse hepatocytes or HSC from CCl_4_-mediated damage or altered gene expression [291]. Hepatocyte EVs bound preferentially to hepatocytes and HSC in vivo [290] and blocked CCl_4_ hepatic fibrosis in mice through their attenuation of hepatocyte damage, pro-inflammatory pathways, HSC activation, and modulation of CCl_4_-regulated hepatic genes [291]. *CRISPR/dCAs9/VP64* in EVs from transfected AML12 mouse hepatocytes was delivered in vitro to mouse HSC in which expression of *dCas9*, *E-cadherin*, and hepatocyte nuclear factor-4α (*HNF-4α*) was increased and expression of *αSMA* and *collagen 1* was decreased, while i.v. administration of the fluorescently-tagged EVs resulted in their localization to mouse liver and were proposed as a fibrosis therapy [292].

#### 7.3.5. EVs from HSC

Quiescent HSC produce EVs that attenuate fibrogenic functions of activated HSCs [37,293,294]. This was attributed to the presence in EVs of miR-199a-5p or miR-214 (or their Twist-1 transcripton factor) which target *CCN2* mRNA and caused suppressed expression of *CCN2* and downstream targets *collagen 1α1* and *αSMA* in activated HSC [37,293,294].

#### 7.3.6. EVs from T Cells, Macrophages or Natural Killer Cells

CD4+ or CD8+T cell microparticles were more abundant in the circulation of patients with chronic hepatitis C (CHC) while microparticles purified from Jurkat T cells utilized CD11a/CD18 to bind to HSC via cellular CD54 (ICAM-1) [295]. This binding resulted in increased expression of fibrolytic genes (*MMP-1, -3, -9, -13*) and this was partly dependent on CD147 (emmprin) and involved activation of ERK1/2 and NFκB pathways [295]. Recent data suggest that the well-characterized elevation of IL-6 levels in NAFLD actually limits the fibrotic response by stimulating macrophage production of miR-223-enriched EVs which are delivered to hepatocytes causing expression of the miR-223 pro-fibrotic target, transcriptional activator with PDZ-binding motif (*TAZ*), to be inhibited [296]. EVs from NK-92MI natural killer cells inhibited TGF-β1-induced proliferation and expression of *αSMA* and *collagen 1α1* in human LX2 HSC in vitro and, when administered to CCl_4_-treated mice, the EVs caused normalization of serum AST/ALT, reduced collagen deposition and attenuation of fibrotic markers [297]. These therapeutic effects were attributed to EV-mediated delivery of miR-223, which targeted *ATG7* and suppressed autophagy in HSC [298].

#### 7.3.7. EVs from Serum

In vitro, serum EVs from normal mice decreased HSC proliferation and fibrotic gene expression and reversed CCl_4_- or ethanol-mediated inhibition of hepatocyte proliferation [299]. Administration of serum EVs to CCl_4_- or TAA-treated mice resulted in reduced hepatic fibrosis, hepatocyte death, inflammatory infiltration, circulating AST/ALT levels and hepatic or circulating pro-inflammatory cytokines whereas serum EVs from fibrotic mice were not therapeutic. MiRs-34c, -151-3p, -483-5p, -532-5p and -687 were enhanced in serum EVs from normal mice as compared to their fibrotic counterparts and inhibited HSC fibrogenesis and hepatocyte injury [299]. Human LX-2 HSC activation was similarly attenuated by serum EVs from healthy human subjects and these EVs contained a similar enrichment of anti-fibrogenic miRs when compared to serum EVs from hepatic fibrosis patients [299].

### 7.4. EVs as Biomarkers in Hepatic Fibrosis

Circulating EVs in experimental or clinical NAFLD or NASH occur at higher frequency and may contain hepatocyte components or markers [243,244,248,249]. In experimental NAFLD, the number of EVs in serum or plasma was correlated with cell death, fibrosis and pathological angiogenesis [248] while F3/F4 patients with NAFLD had frequencies of EVs that were, respectively, increased from plasma platelets or CD31+ leukocytes and decreased from endothelial cells or other leukocyte populations [300]. The association between F3/F4 fibrosis and the liver fibrosis score (LFS) algorithm was improved by inclusion of the CD14+ or CD16+ leukocyte EV frequency, each of which also improved the risk prediction of F3/F4 fibrosis in NAFLD [300].

Serum exosomal miRs discriminated between chronic hepatitis B (CHB), chronic hepatitis C (CHC), NASH and normal individuals while also distinguishing between hepatic inflammation and fibrosis [301]. Progression of hepatic fibrosis in CHC was associated with diminished expression of let-7a and miR- 106b, -1274a, -130a, -140-3p, -151-3p, -181a, -19b, -21, -24, -375, -548l, -93 and -941 and enhanced expression of miR-483-5p and -672-5p [301]. Plasma EVs from F0-F2 HBV or HCV patients showed significant decreases in miR-150, -192, -200b, and -92a levels as compared to healthy controls and these miRs were also downregulated during activation of human HSC in vitro [302]. These miRs did not show the same discriminatory power when measured directly in plasma and were proposed as novel EV markers for early hepatic fibrosis [302]. In CHC, circulating let-7a-5p was correlated negatively with the severity of hepatic fibrosis but not inflammation and had similar efficacy as transient elastography in discriminating liver cirrhosis [303]. Whereas let-7a-5p in serum EVs was also correlated with hepatic fibrosis severity, it was a less powerful measurement than free let-7a-5p. In the same study, miR-122-5p in serum or EVs was correlated with liver damage but was not correlated with hepatic fibrosis or inflammation [303]. Serum exosomal miR-103-3p levels allowed discrimination between HBV patients with no, mild or severe hepatic fibrosis, consistent with the role of exosomal miR-103 in macrophage-mediated activation of HSC (see above) [270]. Plasma EVs from CCl_4_-treated rats contained upregulated mir-122, -99b, and -192 and downregulated miR-100 and these were variably differentially expressed when the rats were treated with anti-fibrotic phosphodiesterase 5 inhibitor supporting their potential as biomarkers during fibrosis therapy [304]. Finally, in Schistosoma infection, levels of serum exosomal miR-92a-3p, 146a-5p and 532-5p discriminated patients with grades I-III hepatic fibrosis from those with no hepatic fibrosis, while exosomal miR-146a-5p also showed promise for discriminating grades 0–I (mild) hepatic fibrosis from grades II–III (severe) hepatic fibrosis [305].

## 8. Pancreatic Fibrosis

### 8.1. Causes and Pathological Features of Pancreatic Fibrosis

Pancreatic fibrosis is a major pathophysiological feature of chronic pancreatitis or desmoplastic stroma in pancreatic ductal adenocarcinoma (PDAC) [306,307]. Pancreatitis is often associated with chronic alcohol consumption and results in acinar cell injury and protease-driven autodigestion. The acute form of the disease involves episodic pancreatic inflammation which, if severe and continuous, can lead to chronic pancreatitis which is characterized by progressive acinar cell destruction, extensive perilobular and intralobular fibrosis, and reduced exocrine or endocrine function leading to, respectively, maldigestion or diabetes [307]. The fibrotic component reflects the unabated matrigenic actions of pancreatic stellate cells (PSC) which reside in peri-acinar and peri-ductular locations and are functionally similar to HSC in the liver [308,309]. PSC also play a central role in the development of desmoplasia in PDAC, an extremely aggressive disease in which most patients present with non-resectable tumors at the time of diagnosis [306,308]. A major histopathological feature of PDAC is an abundant interstitial stroma comprising fibroblasts, myofibroblasts, inflammatory cells and PSC and an extensive ECM that may constitute 80% of the tumor mass and which functions to sustain tumor growth and increase its invasive potential [310,311].

### 8.2. Mechanistic Aspects of EVs in Pancreatic Fibrosis

In chronic pancreatitis in mice, miR-21 expression was upregulated in PSC and participated with CCN2 in cellular or EV positive feedback loops to drive collagen expression [36]. When added to pancreatic cancer cells, exosomes from PSC or cancer-associated fibroblasts caused increased cancer cell aggressiveness, proliferation, migration, EMT, activation of ERK1/2, AKT, and expression of CXCL1/2, in part due to the actions of exosomal miR-21, -451a, and -5703. [312,313,314,315]. On the other hand, exosomes from pancreatic cancer cells promoted PSC proliferation, migration, fibrogenesis and recruitment, due to activation of a Lin28B/let-7/HMGA2/PDGFB axis in PSC by exosomal lin28 protein [316] and stimulation of PSC fibrogenesis by miR-1290 [317]. Thus PDAC progression and desmoplasia are driven by bi-directional EV transfer between cancer and stromal compartments.

## 9. Skin Fibrosis

### 9.1. Causes and Pathological Features of Skin Fibrosis

Healing of skin wounds involves coordinated interaction between multiple cell types (keratinocytes, fibroblasts, endothelial cells, immune cells). Epidermal re-epithelialization is generally achieved at high fidelity but macrophage recruitment into the dermis is a trigger for (usually limited) scarring due to activation and matrigenesis in wound fibroblasts and myofibroblasts [318]. This response is exaggerated and modified in hypertrophic scars and keloids which lack effective treatments and cause discomfort, functional disability, and psychological distress [319]. Hypertrophic scars are often a disfiguring result of surgery or trauma (e.g., burns) and contain an abundance of myofibroblasts, oriented collagen bundles, and regress partially, while keloids contain thick collagen bundles, few fibroblasts, extend beyond the wound margin, do not regress, have occluded blood vessels, and are more common in dark-skinned individuals [320,321,322].

Skin fibrosis can also occur in immune-related disease processes. For example, systemic sclerosis (SSc) or scleroderma is a rare life-threatening systemic autoimmune disease with a highly dysregulated immune response, endothelial damage, fibroproliferative vasculopathy, and progressive fibrosis in the skin and numerous internal organs [323]. Depending on the extent of skin involvement, SSs is clinically classified as a limited form associated with pulmonary arterial hypertension or a diffuse cutaneous form that is associated with pulmonary fibrosis [323]. As another example, inflammation and fibrosis of multiple organs (skin, mucosa, lungs) is a feature of chronic graft versus host disease (cGVHD) which typically occurs in long-term survivors of allogeneic hematopoietic stem cell transplantation to treat hematologic malignancy [324]. cGVDH patients face disablement due to fibrosis of the skin and joints as well as the life-threatening consequences of pulmonary fibrosis [324].

### 9.2. Mechanistic Aspects of EVs in Skin Fibrosis

In SSc patients with lung fibrosis, there was a marked increase in annexin V^−^ endothelial cell-derived EVs in plasma which was associated with diffuse SSc [325]. In vitro, SSc fibroblasts expressed higher levels of EV-associated tetraspanins and produced more EVs than normal fibroblasts [326]. SSc fibroblast EVs stimulated collagen production in normal fibroblasts and contained higher levels of miR-142-3p and lower levels of miR-150a and -196a [326]. Serum EVs from SSc patients also contained higher tetraspanin levels than in control patients [327] but serum EV concentrations were reduced in SSc patients, possibly due to impaired EV transport from skin to bloodstream due to SSc-associated vasculopathy [326]. Vasculopathy and microvascular fibrosis were linked to inhibition of endothelial cells by serum- or neutrophil-derived EVs, possibly mediated by S100 calcium binding proteins [327]. As compared to control patients, serum EVs from patients with diffuse SSc were enriched with a profibrotic transcriptome, a subset of which was shared with serum EVs from limited SSc; these cargo differences were reflected in their relative in vitro fibrogenic actions [328]. Similarly, EVs purified from cultured peripheral blood neutrophils from diffuse SSc patients contained 22 differentially regulated miRs and 281 differentially regulated lncRNAs that were associated with fibrosis-related signaling by *Wnt*, *AMPK*, *Il-23* and *Notch*, with concomitant changes in these pathways being induced in EV-treated endothelial cells or fibroblasts [329]. Finally, EVs from wound myofibroblasts contained FGF2 and VEGF and promoted proliferation of dermal fibroblasts, wound myofibroblasts, and endothelial cells [330]. In vitro, serum or plasma stimulated production of EVs by wound fibroblasts but not by dermal fibroblasts or fibroblasts from hypertrophic scar, suggesting that production of myofibroblast EVs is dynamically regulated by myofibroblast phenotype [330].

### 9.3. Therapeutic Actions of EVs in Skin Fibrosis

#### EVs from Adult Stem Cells

BM-MSC produced EVs that were therapeutic for systemic cGVHD in mice, including anti-fibrotic effects in the skin, lung, and liver [331]. Principal therapeutic effects included correction of cGVDH-associated alterations in T cell immune responses [331]. Umbilical cord-derived MSC EVs accelerated wound closure and reduced scar formation and myofibroblast frequency in full thickness skin wounds in nude mice and also reduced TGF-β-induced *αSMA* and proliferation, migration and contraction in fibroblasts in vitro [332]. These actions were linked to exosomal miR-21, miR-23a, miR-100, miR-125b, and miR-145 which targeted components of the *TGF-β-Smad2 axis* [332]. These EVs also attenuated skin fibrosis in a mouse model of cGVHD by reducing macrophage frequency and expression of *TGF-β/Smad2* while also modulating B cell-mediated immune responses [333].

When added to fibroblasts in vitro, EVs from human AECs promoted proliferation, wound migration, and expression of MMP1 while decreasing expression of *collagen I* and *III* [334]. In vivo, AEC EVs reduced scar formation in full thickness skin wounds in rats by promoting more rapid would closure and improving collagen organization [334]. Similar accelerated healing occurred in full thickness wounds in mice that were treated with mouse serum exosomes, although collagen expression was not significantly altered [326].

## 10. Perspective and Concluding Remarks

Considerable evidence has accumulated over the last few years, which supports the notion that fibrosis is an EV-regulated pathology in multiple organs. Regarding the role of EVs that are instrinsically produced during fibrosis, the principal findings are that cellular transdifferentiation into myofibroblasts or the activation of myofibroblasts and their production of fibrosis-related signaling molecules or components (e.g., TGF-β, CCN2, PDGF, αSMA, FN, collagen, tissue inhibitors of MMPs), are stimulated by EVs that derive from cells (or fluids) in the vicinity of the fibrosing injury, or that are produced by these cells in vitro when they experience damage or stress or in response to environmental or molecular cues that promote migration, infiltration or activation. These direct pro-fibrogenic effects are elicited by EVs from many different cell types that play an orchestrated role in wound repair responses including epithelial cells, macrophages, neutrophils, T cells, B cells, interstitial fibroblasts, myofibroblasts and endothelial cells as well as by endothelium-associated cells such as pericytes, podocytes and HSC. Even so, many conclusions are based on in vitro observations in which either purified EVs from a presumptive EV donor cell type are investigated for their effects on fibrogenic readouts in cultured myofibroblasts or co-culture approaches are used to provide evidence of functional EV-mediated interactions between injured or damaged donor cells and recipient fibrogenic cells. Currently, there is a lack of compelling evidence that these intrinsic EV pathways exist in vivo as de facto drivers of fibrosis and proof for this will necessitate technically challenging approaches that involve cell-specific interruption of EV biogenesis, cargo loading, delivery or targeting in animal models of fibrosis. While definitive answers to this question will likely require the use of genetically modified animals in which EV production, trafficking or responses are altered, support for intrinsic EV-mediated regulation of fibrosis has come from studies demonstrating fibrosis progression by injury-related EVs in vivo [68,72,109,170,179,182,251,257,258,259,260] and by the suppression of experimental renal or cardiac fibrosis in vivo by chemical- or drug-induced attenuation of EV secretion or miR content. [108,109,174,185]. A further mechanistic complication is that fibrosis is preceded by tissue injury and inflammation and the involvement of EVs in any one of these processes may be permissive for downstream pro-fibrotic pathways. Indeed, EVs from damaged or effector cells can exacerbate parenchymal dysfunction as well as promote infiltration and/or activation of immune cells and macrophages and these are likely important mechanisms, albeit indirect, by which a fibrotic environment is favored or primed by injury-related EVs. That said, EVs drive fibrosis by regulating a broad variety of well-characterized fibrosis-associated signaling pathways and several of these such as Wnt/β-catenin, Notch, YAP/TAZ, PTEN, AKT, PPAR, HIF, NF-κB, CXCR/CXCL, and MAPK/ERK are implicated in EV pro-fibrotic actions across multiple organ systems (Table 1).

With respect to therapeutic aspects, there is abundant evidence that experimental fibrosis in many different organ systems is attenuated by EVs from numerous varied sources. As well as direct targeting of pro-fibrotic factors such as TGF-β, CCN2, PDGF, and TIMPs, the actions of therapeutic EVs in multiple organs arise due to their modulation of well-characterized fibrosis-associated signaling pathways (Table 1). Even so it is very apparent that in vivo anti-fibrogenic effects in myofibroblasts by therapeutic EVs frequently occur in conjunction with broader therapeutic effects including improved scores for cell injury, inflammation and immune function. Thus anti-fibrotic effects of EVs may arise through their targeting of upstream pathways in other injury-related cell types rather than (or in addition to) myofibroblastic fibrogenesis and this question has only been addressed in a few studies, for example by delaying EV administration until inflammation has waned and fibrosis is well-established. Furthermore, demonstration of anti-fibrogenic actions in vitro does not preclude more complex EV behavior in vivo. More studies are needed to understand the mechanistic basis of anti-fibrotic EVs by establishing their full repertoire of cellular interactions and targets after injury. With these caveats in mind, a plethora of exciting and innovative strategies have been used to investigate EV-based anti-fibrotic therapy. Successful therapeutic outcomes in experimental organ fibrosis have been obtained using EVs from a strikingly broad slate of cell types including non-injured parenchymal cells, fibroblasts, stem cells, macrophages, immune cells, and endothelial cells as well as from body fluids such as serum or plasma. The anti-fibrotic actions of EVs from stem cells are of particular interest because they offer a potentially safer alternative to their producer stem cells which are themselves therapeutic in many diseases and fibrotic conditions but are associated with a potential for tumorigenesis and low rates of engraftment which reduces therapeutic efficacy [335]. Stem cell EVs have unequivocally emerged as being broadly applicable for treatment of many types of organ fibrosis irrespective of the stem cell source (Table 2) and it is quite possible that stem cell EVs will have utility as cell-free pan-organ anti-fibrotics. Since many of the studies to date have involved EV effects on a wide variety of different and sometimes disparate signaling pathways, future studies will need to focus on whether EV anti-fibrotic actions across different organs occur as the result of the regulation of shared targets by the same EV cargo components.

Additionally, evidence has accumulated demonstrating an intimate association between fibrosis-associated changes in autophagy and the production or action of pro-fibrotic EVs, consistent with the recognition that these vesicular transport systems pathways are functionally linked [337,338,339]. Autophagy involves the lysosomal degradation of stress-induced defective proteins and organelles after they have been encapsulated in autophagosomes, a process that contributes to tissue homeostasis and which is altered during fibrosing injury. When autophagy is compromised, exosome release may become an alternative mechanism of removing stress-induced components from the cell that may have pro-pathogenic effects in other cells For example, in fibrotic liver, the levels of the stress protein TRIB3 were increased in hepatocytes causing impaired autophagy and obstruction of autophagic flux of SQSTM1/p62 as well as triggering the production of inhibin/activin A-enriched exosomes that stimulated HSC activation [252]. Similarly, in a model of PDGF-mediated HSC activation, autophagy was decreased with a concomitant increase in the release of pro-fibrogenic exosomes and microvesicles due to, respectively, reduced MVB degradation and stimulation of ROCK1 activation [262]. On the other hand, anti-fibrotic effects of EVs in several organ systems have been attributed to, or are coincident with, alterations in autophagic pathways in parenchymal and/or fibrogenic cells. For example in the liver, AD-MSC EVs enriched in either miR-181-5p or *mmu_circ_0000623* were anti-fibrotic and also stimulated autophagy, with the latter involving activation of a miR-125-*ATG4D* axis in hepatocytes [277,278]. In contrast, autophagy was reported to be increased during HSC activation [340] but the targeting of *ATG7* by miR-223 delivered in NK cell exosomes resulted in reduced HSC autophagy, thereby suppressing HSC activation [298]. In stz DN, EVs from BM-MSC were anti-fibrotic in the kidney but concomitantly induced autophagy and increased the expression of autophagy markers *LC3* and *beclin-1* while decreasing the expression of *mTOR* [126]. In cardiac MI models, EVs from iPSC-derived cardiomyocytes were anti-fibrotic and promoted myocardial repair and autophagy [219] but the reparative and anti-fibrotic affects EVs from BM-MSC were associated with decreased expression of *LC3B,* and EV therapy was enhanced by enrichment with miR-101 which blocks autophagy [185]. Although current data are not fully consistent as to the nature and direction of dynamic alterations of autophagy during fibrosing injury (which may reflect injury severity/type or cell-specific or temporal differences between the experimental systems), it is nonetheless evident that changes in autophagosome function and autophagic flux influence the production of EVs and their pathogenic payloads as well as providing a target for EV therapy.

While miRs and, to a lesser extent, proteins have generally been implicated in the pro- or anti-fibrotic actions of EVs, there is uncertainty as to the relative contribution of these components in eliciting meaningful biological responses. A role for a specific EV miR is often inferred when its ablation or enrichment in EVs by experimental manipulation of the producer cells results in altered expression of its presumed target in recipient cells, but such manipulation likely results in additional downstream miR-dependent changes in gene expression in the producer cells that are also manifested in the EVs, such that altered outcomes in the recipient cells may be due to other altered EV cargo molecules. Co-culture of EV producer cells with recipient cells that harbor untranslated region (UTR) reporters has also been used to demonstrate that miR circuitry in EVs is biologically effective at intrinsic levels of EV production but the high sensitivity of UTR reporter assays may not recapitulate the conditions needed for biologically relevant altered gene expression. Moreover, it has been argued both that the intra-vesicular concentration of many miRs may be too low to result in delivery of an effective dose to target cells and that the structural conformation of mature miRs in EVs may not ensure their functional activity in recipient cells [341,342]. Although these aspects are unresolved and contrary to many studies that have implicated miR-based mechanisms of EV action, more detailed consideration needs to be given to the role of other EV constituents in regulating fibrosis. With respect to functional EV proteins, therapeutic actions of MSC EVs in reducing myocardial infarct after I/R injury have been attributed to combinatorial actions of EV enzymes [341], while the proteome of EVs from activated HSC is consistent with their ability to promote fibrogenesis and matrigenesis in the liver [267]. While more data will undoubtedly be obtained in the future using reductionist approaches to establish the functional role of individual EV components, a molecular systems strategy that integrates the entire vesicle payload (miR, RNA, protein, lipid) [343] will likely be needed to comprehensively account for the pro- or anti-fibrotic actions of a specific EV population.

Although the biomarker potential for EVs in certain stages of chronic diseases is high, more data are needed to determine if EV components are present at sufficient sensitivity and specificity to allow them to be used diagnostically or prognostically for accurate detection and staging of fibrotic pathology against a background of other pathological sequalae. Emerging evidence does however suggest that body fluids (serum, plasma, lung fluid, urine) harbor EV populations that undergo dynamic changes in frequency or compositions of their molecular payloads that are associated with fibrotic changes. The principal cargo components identified to date as promising biomarker candidates are various miRs which undergo alterations in expression that are “pro-fibrogenic” and are often reflective of their known function or expression in fibrotic tissues. Although the EV miRs so far characterized are often distinct between different fibrotic organ systems, they are frequently associated with inflammation, production of myofibroblasts, and collagen production. On the other hand, miR-21 and several members of the let-7 family have been identified as potential biomarkers for fibrosis of the lung, kidney, heart, and liver [95,96,162,173,231,301,344] indicating that these miRs may have shared pan-organ functions or mechanisms of production during fibrosis. Although other EV components are less well studied, evidence from renal fibrosis suggests that some EV mRNAs (e.g., *CD2AP*) and proteins (E-cadherin, vimentin) may also be useful fibrotic markers. While data so far support the use of EV components of as fibrosis biomarkers, this aspect is in its infancy and more rigorous analysis is needed to clarify this aspect of EV biology. In the long run, EV biomarkers will most likely be impactful clinically by complementing other patient tests (serum chemistry, imaging, etc.) rather than being used as a stand-alone tool for fibrosis assessment.

## Figures and Tables

**Table 1:** Representative examples of fibrosis-related signaling pathways or components that are affected by pathogenic or therapeutic EVs in different organ systems.

EV-Targeted Signaling Component	Lung		Kidney		Heart		Liver		Pancreas	Skin
	Fibrosis	Therapy	Fibrosis	Therapy	Fibrosis	Therapy	Fibrosis	Therapy	Fibrosis	Fibrosis
Wnt/β-catenin	Martin-Medina et al., 2018 [66]		Wu et al., 2017 [118]		Ibrahim et al., 2019 [210]			Rong et al., 2019 [273]		Li et al., 2020 [329]
	Xie et al., 2020 [67]				Dzialo et al., 2019 [177]					
	Parimon et al., 2019 [68]				Cai et al., 2020 [179]					
	Zhang et al., 2020 [82]									
SIRT-1/-3	Kadota et al., 2020 [70]	Chen et al., 2020 [108]								
PDL1	Kang et al., 2020 [71]									
Notch	Kadota et al., 2020 [70]			He et al., 2020 [131]		Xuan et al., 2020 [204]				Li et al., 2020 [329]
	Wang et al., 2021 [78]									
YAP/TAZ	Wang et al., 2021 [78]			Ji et al., 2020 [137]			Hou et al., 2020 [296]			
PTEN			Zhou et al., 2013 [110]		Xia et al., 2018 [173]	Sun et al., 2020 [187]				
						Kang et al., 2019 [230]				
AKT			Zhou et al., 2013 [110]		Xia et al., 2018 [173]	Shao et al., 2020 [184]	Wang et al., 2015 [269]		Li et al., 2020 [314]	
						Sun et al., 2020 [187]				
PPAR			Liu et al., 2020 [112]				Povero et al., 2015 [246]	Rong et al., 2019 [273]		
HIF	Wang et al., 2021 [78]		Liu et al., 2020 [112]	Zou et al., 2016 [139]		Sun et al., 2020 [186]		Wan et al., 2019 [264]		
Dyrk2					Yang et al., 2018 [170]					
Nrf2					Tian et al., 2018 [176]					
Neat 1					Kenneweg et al., 2019 [171]					
HuR					Govindappa et al., 2020 [180]					
Foxo3a					Wang et al., 2020 [182]					
HSP70,72					Yang et al., 2019 [183]	Feng et al., 2014 [203]		Alhomrani et al., 2017 [283]		
								Ohara et al., 2018 [284]		
NF-κB		Xiao et al., 2020 [84]					Cannito et al., 2017 [247]			
							Khodayari et al., 2020 [271]			
NLRP3		Sun et al., 2019 [91]					Cannito et al., 2017 [247]			
ROCK		Shi et al., 2020 [128]				Xuan et al., 2019 [220]	Hirsova et al., 2016 [244]			
							Lee et al., 2017 [245]			
							Povero et al., 2015 [246]			
TLR							Eguchi et al., 2020 [250]			
JAK/STAT							Devhare et al., 2017 [255] Khodayari et al., 2020 [271]	Qu et al., 2017 [277]		
KLF4							Chen et al., 2020 [270]			
CXCR/CXCL							Khodayari et al., 2020 [271]	Du et al., 2020 [276]	Takikawa et al., 2017 [313]	
KIT							Kim et al., 2018 [272]			
SNAIL				Zhang et al., 2020 [138]						
Bcl2						Zhao et al. (2015) [195]		Qu et al., 2017 [277]		
MAPK/ERK				Liu et al., 2020 [136]	Yuan et al., 2018 [225]		Korneck et al., 2011 [295]		Ma et al., 2020 [315]	

**Table 2:** Anti-fibrotic properties and actions of stem cell EVs.

EV Source	Organ Targeted	Fibrosis Model	Principal EV Effects	EV Features Associated with Therapy	Reference
Bone marrow MSC	lung	bleomycin	Reversal of septal thickening and fibrosis, including late fibrotic stages lacking inflammation Promotion of anti-inflammatory immunoregulatory monocytes or macrophage phenotypes in lung and bone marrow.	Targeting of *FZD6* by miR-29b-3p	[79]
			Decreased fibrosis, hydroxyproline in lung tissue		[83]
		hyperoxia-induced BPD	Improved lung function, decreased fibrosis, vascular remodeling, reversal of pulmonary hypertension Stimulation of M2 macrophages and corrected expression of genes involved in immunity and inflammation		[80]
		LPS-induced acute lung injury	Suppressed αSMA, TGF-β, and *Col I & III* Suppressed p65, IKKβ, p-IKBα, p-IKBβ, Shh	Inactivation of *NFκB* and *Hh* pathways by EV miR-182-5p and miR-23a-3p	[84]
	kidney	Stz DN	Correction of interstitial fibrosis, tubular expansion, TEC vacuolation, atrophy and *TGF-β* expression		[125]
			Decreased mesangial expansion, collagen deposition, TGF-β production, glomerular basement membrane thickening Increased LC3, beclin-1, decreased mTOR & fibrotic markers Induction of autophagy		[126]
			Restoration of renal function, reduced glomerular basement membrane thickening, glomerular hypertrophy Reduced tubulointerstitial fibrosis and expression of fibrosis-associated genes	EV miRs predicted to target *TGF-β*, *EGFR, PDGFR, ARF-6, mTOR*, *VEGF,* p53-apoptosis, *ZTM, TNF* pathways	[127]
		AA CKD	Restored kidney function Reduced tubular necrosis, fibrosis and CD45+ cells Reduced expression of *collagen1α1, TGFβ1, αSMA* Downregulated miR-21, -34, -132, -342, -212, -214; Upregulated miR-194, -192, -378a		[143]
		UUO	Reduced tubular dilation, interstitial expansion, collagen deposition, inflammatory cell infiltration, expression of *Kim-1*, *collagen IVα1, TGF-β1, TGF-βR1*	EVs were experimentally augmented with miR-Let7c	[130]
			Reduced fibrosis, inflammation, oxidative stress, apoptosis	Inhibition of Rho/ROCK by EV MFG-E8	[128]
	heart	MI	Reduced cardiac fibrosis Reduced cardiomyocyte apoptosis in vitro due to suppression of *Mecp2* by miR-22	EVs were from miR-22-transfected MSC since ischemic conditioning of MSC led to elevated miR-22 in EVs.	[191]
			Improved heart function (ejection fraction, fractional shortening) and vascularity. Decreased infarct size, fibrosis, and cardiomyocyte apoptosis; increased recruitment of CPC	EVs were from ischemic conditioned MSC and contained elevated miR-210. Cardioprotective effects were associated with miR-210	[192]
			Improved heart function and vascularity Decreased apoptosis, inflammation, inflammatory cytokines, and fibrosis/infarct size M1 macrophages decreased, M2 macrophages increased	EVs were experimentally augmented with Lamp2b-IMPT to target ischemic myocardium	[188]
			Decreased infarct size, fibrosis, and expression of TGF-β or collagen I Improved heart function Increased M2 macrophage frequency, reduced serum pro-inflammatory cytokines Decreased expression of *LC3B*	EV therapy enhanced by enrichment with miR-101 which blocks autophagy	[185]
			Improved survival of EV-preconditioned CSC Improved angiogenesis and cardiac function, reduced fibrosis	EV treatment of CSC before CSC therapy. In CSC, EVs caused increased expression of genes associated with angiogenesis, heart development, cell proliferation, migration and differentiation	[193]
			Preserved cardiac function, enhanced angiogenesis, reduced fibrosis	EVs were experimentally augmented with HIF-1α	[186]
			Improved endothelial function and cardiomyocyte survival Decreased fibrosis	EV miR-221-3p inhibited *PTEN* leading to stimulation of AKT and suppression of apoptosis	[187]
		Pressure-overload	Reduced cardiac hypertrophy, myocardial apoposis, and fibrosis	EV-mediated senescence in cardiac myofibroblasts	[190]
	liver	CCl_4_	Enhanced LFTs and hepatocyte regeneration Reduced fibrosis, inflammation and oxidative stress Inhibition of Wnt/β-catenin pathway		[273]
	skin	cGVDH	Improved survival and pathology score Reduced fibrosis in skin, lung, liver Modulation of effector T cell populations, suppressing Th17 cells and inducing Treg		[331]
Adipose tissue MSC	lung	Particulate inhalation	Reduced apoptosis and necrosis in Type II alveolar cells Suppressed ROS, inflammation, fibrosis	Suppression of *TGF-βR1* by EV let-7d-5p	[87]
	kidney	Metabolic syndrome + renal artery stenosis	Attenuation of renal inflammation and fibrosis, improved medullary oxygenation Restoration of kidney function (kidney volume, RBF, GFR, serum creatinine)	EV IL-10 mRNA expressed 100x higher than in producer cells. EVs non-therapeutic upon knockdown of IL-10 in MSC.	[132]
			Reduced peritubular capillary loss and fibrosis	EVs from GDNF-transfected AD-MSCs; enhanced *SIRT-1* and *eNOS* in endothelial cells	[108]
			Increased M2 macrophages and anti-inflammatory CD8+ T cells, decreased M1 macrophages and oxidative stress	EV TGF-β associated with anti-inflammation; EV mitochondrial-regulating miRs associated with reduced oxidative stress	[133]
		UUO	Suppressed oxidative stress and apoptosis and increased proliferation in TEC Reduced fibrosis	EV delivery of casein kinase 1δ and E3 ubiquitin ligase β-TRCP, targeting *YAP* and *collagen*	[136,137]
	kidney & heart	DOCA-salt hypertension	Preserved kidney filtration and suppressed renal inflammation, fibrosis, and expression of *αSMA, desmin, collagens 1α1 & 4α1, and FN1, CD68, PAI1, MCP-1, IL-6* Suppression of cardiac fibrosis and maintained blood pressure Regulation of renal EMT via miT-200/*TGF-β*		[135]
	heart	MI	Attenuated cardiac apoptosis and fibrosis, restoration of heart function Suppression of Il-1β, IL-6, TNF-α, IFNγ and promotion of M2 macrophage phenotype Stimulation of S1P/SK1/S1PR1 causing suppressed NF-κB and TGF-β		[199]
			Reduced cardiac infarct size, fibrosis, cardiomyocyte apoptosis, and serum IL-1β, Il-6, TNF-α Improved microvascular density Greater therapeutic outcomes for miR-126-enrcihed versus control EVs	EVs were experimentally augmented with miR-126	[200]
			Suppression of apoptosis, inflammation, fibrosis and serum IL-1β, Il-6, TNF-α Targeting by miR-146a of early growth response factor 1 and downstream *TLR4/NF-κB*.	Evs were experimentally augmented with miR-146a	[201]
			Improved cardiac function and vascularity Decreased inflammation and fibrosis Encapsulated EVs not advantageous	EVs administered in free form or encapsulated within peptide hydrogel	[189]
			Decreased infarct size, fibrosis, and infiltration of CD68 inflammatory cells	As compared to producer MSC, EVs had increased miR-29 or -24 and decreased miR-15, -21, -34, -130, or -378	[336]
	liver	CCl_4_	Suppression of HSC activation and collagen deposition miR-122 targeting of *IGF1R, cyclin G(1) and prolyl-4-hydroxylase α1*	EVs were experimentally augmented with miR-122	[275]
			suppression of fibrosis, collagen I, vimentin, αSMA and FN miR-181 targeting of Stats3 and Bcl2 and stimulation of autophagy	EVs were experimentally augmented with miR-181-5p	[277]
			reduced hepatic vimentin, FN, collagen, and CXCL1 reduced fibrosis and collagen volume reduced serum TNF-α, IL-6, IL-17, AST, ALT and TB	EVs were experimentally augmented with miR-150-5p which targeted *CXCL1*	[276]
			reduced αSMA and collagen stimulated production of autophagy-related proteins	EVs were experimentally augmented with *mmu_circ_0000623* which targeted miR-125/*ATG4D*-mediated autophagy	[278]
Tonsil-derived MSC	liver	CCl_4_	improved liver morphology, restoration of serum ALT and AST and hepatic G6pc mRNA reduced fibrosis reduced hepatic *TGF-β, αSMA, vimentin, collagen 1α1,* hydroxyproline	EVs enriched in miR-486.5p which suppressed Hh signaling in HSC by targeting the Smo Hh receptor	[281]
Liver stem cells	kidney	AA CKD	Reduced tubular necrosis, fibrosis, and CD45 cell infiltration. Modulation of miRs affecting fibrosis (*Wnt*, inflammatory cytokines, *PDGF, GRG, TGF-β*) and downregulated expression of 14 pro-fibrotic genes		[142]
	liver	NASH	Restored serum ALT Reduced fibrosis and CD45 inflammatory cells Corrected expression of 28/29 genes associated with fibrosis or inflammation		[285]
		CC_l4_	Correction of serum ALT, oxidative stress, and apoptosis Reduced hepatic collagen and hydroxyproline	EV effects were substantially improved by co-administration of Nilotinib	[286]
		MDR2-/- primary sclerosing cholangitis	Reduced ductular reaction and biliary fibrosis Reduced chonangiocyte growth and HSC activation Reduced hepatic or cholangiocyte expression of let-7 targets (*Lin28a/b,IL-13, NR1H4, NF-κB*)	EVs enriched in let-7	[287]
Amnion epithelial cells	lung	bleomycin	Reduced inflammation, fibrosis and EMT increased frequency of lung progenitor cells	EV enriched with fibrosis-related miRs (PI3-AKT, MAPK, Ras, Hippo, TGF-β, FAK) and proteins associated with apoptosis, developmental growth, MAPK, inflammation, EGF, PDGF, FGF.	[89]
		Bleomycin or OVA/NA	Reduced inflammation and neutrophil or macrophage infiltration Reduced fibrosis and subepithelial ECM Modulation of epithelial damage, *TGF-β* expression and EMT Increased dynamic lung compliance	Some EV effects augmented by serelaxin	[88]
	liver	CCl_4_ fibrosis	Reduced fibrosis and number of activated HSC Reduced macrophage frequency with M2 phenotype predominating,	EVs contain MFGE8, HSP72, SOD1 which suppress TGF-β signaling	[283]
		NASH	Decreased frequency of KCs and expression of *TNF-α, IL-1β, IL-6, TGF-β* Suppressed fibrosis and activation of KC and HSC Suppressed TLR4 signaling		[284]
	skin	Full thickness wounds	Increased rate of wound healing and wound closure, reduced scar area Promotion of fibroblast proliferation, differentiation, migration and collagen production		[334]
Lung spheroid cells	lung	Silicableomycin	Preserved lung architecture and partial restoration of lung function Decreased fibrosis, collagen deposition, apoptosis, alveolar epithelial injury, vascular injury Decreased lung *Smad3* expression and circulating MCP-1	EVs enriched for miR-30a, let-7, miR-99	[90]
Cardiosphere-derived cells CDC	kidney & heart	Ang II	Attenuation of cardiac hypertrophy, inflammation, fibrosis Attenuation of renal function, inflammation, fibrosis Modulation of systemic and tissue IL-10	Therapeutic effects mimicked by EV-FF1, the most abundant small RNA constituent in CDC EVs	[145]
	heart	MI	Improved cardiac function, decreased fibrosis and scar mass, increased infarct wall thickness and microvessel density Comparable therapy using CDC EV-primed fibroblasts	EVs enriched in miR-146a-5p, 302b-3p, 181b-5p, 155-5p	[207]
			Preserved cardiac function Decreased scar size, ventricular collagen, and cardiomyocyte hypertrophy Increased microvessel density		[208]
		Pediatric dilated cardiomyopathy	Improved cardiac function, decreased fibrosis	Antifibrotic effects associated with suppression of *TRAF6/Smad4/FOS* by EV miR-146a-5p	[209]
		Doxorubicin	Improved cardiac function Decreased fibrosis and apoptosis Increased cardiomyocyte proliferation and angiogenesis	Therapy improved by conjugation of cardiac homing peptide to EV	[209,211,212]
Cardiac MSC (Sca-1+)	heart	MI	Decreased fibrosis Increased angiogenesis and microvessel density in infarct area Decreased apoptosis of endothelial cells, and cardiomyocytes	EVs enriched in proteins associated with ECM, adhesion, collagens, integrins. EVs from Notch1-overexpressing C-MSC were tested therapeutically	[204]
Cardiac resident progenitor cells	heart	Doxorubicin/trastuzumab	Reversal of left ventricular dysfunction, fibrosis, CD68 inflammatory cell infiltration, and iNOS expression. EV therapy blocked by silencing of EV miR-146-5p which targeted *Traf6* (inflammation) and *Mpa* (cell death)	EVs were purified from CRPCs derived from outgrowths of primary atrial tissue explants. EVs enriched in redox-regulating proteins and miR-146-5p	[205]
		MI	CRPCs implanted subcutaneously were therapeutic for ventricular function, fibrosis, cardiomyocyte hypertrophy and microvessel density EVs liberated from these CRPCs trafficked to heart (myocardium interstitium, perivascular, cardiomyocytes), skeletal muscle, liver, lung, kidney	W8B2^+^ CRPCs were obtained from outgrowth of primary atrial tissue explants. EVs contained proteins associated with inflammation, immunoregulation, tissue remodelling, fibrosis.	[206]
Umbilical cord-derived MSC	lung	Hyperoxia-induced BPD	Decreased inflammation, fibrosis, and vascular defects Stimulation of M2 macrophages, altered immune/inflammation gene expression, improved lung function Decreased pulmonary hypertension, improved exercise capacity	EVs isolated from Wharton’s jelly	[81]
		Monocrotaline	Restored cardiac function, attenuated fibrosis, vascular remodeling	*Wnt5a/BMP* regulated by Wharton’s jelly EVs	[82]
		silicosis	Alleviation of respiratory resistance, airway resistance, tissue dampening Inhibition of fibrosis		[85]
	kidney	I/R AKI	Suppression of tubular necrosis, tubular dilation, apoptosis and fibrosis, stimulation of cell proliferation and angiogenesis normalization of serum creatinine & blood urea nitrogen Reduced HIF-1α expression, increased VEGF	VEGF delivered directly by EVs and insensitive to RNase. All other effects abrogated by RNase treatment of EVs	[139]
			Suppression of kidney damage, fibrosis, and apoptosis; stimulation of cell proliferation, preserved renal function Therapy enhanced by EVs from Oct-4-overexpressing donor cells and reduced by EVs from cells in which oct-4 was inhibited Reduced renal expression of *Snail*, an EMT-inducer that is a target of oct-4	Delivery of EV oct-4	[138]
	heart	MI	Improved heart function, reduced fibrosis, reduced cardiomyocyte apoptosis	EVs regulate *Bcl-2* expression	[195]
			Reduced inflammation (CD68 cell infiltration, serum TNF-α), fibrosis, collagen deposition, TGF-β expression, apoptosis, improved angiogenesis	EVs encapsulated in functional peptide hydrogel	[196]
			Restoration of cardiac function, reduced fibrosis and inflammation miR-24 targets pro-apoptotic *Bim*	EVs more effective when purified from cells lacking human leuckocyte antigen light chain β2-microglobulin (B2M) to avoid T cell immunity EV enriched in miR-24	[184]
			Improved cardiac function, increased cardiac vascularity, reduced fibrosis	Targeting of apoptotic peptidase activating factor by EV miR-136	[197]
		Type 2 diabetes stroke	improved cardiac function, decreased fibrosis, *TGF-β* expression, M1 macrophages, oxidative stress, capillary density increased cardiac expression of miR-216 and decreased expression of its targets, *Spred-1, MCP-1, VCAM1*		[198]
		Metabolic syndrome + renal artery stenosis	increased density of cardiomyocytes and capillaries reduced inflammatory cytokine expression, reduced fibrosis		[202]
	liver	CCl_4_	Reduced fibrosis, surface fibrous nodules, inflammation Suppression of serum AST, HA, and TGF-β Suppression of TGF-β and myofibroblast production		[280]
			Reduced fibrosis, inflammation, and tumor growth Suppression of collagen expression and oxidative stress		[279]
	skin	Full thickness wound	Reduced scar formation and myofibrblast frequency EV miR-mediated suppression of TGF-β/Smad2	EVs enriched in miR-21, 23a, 100, 125b, 145	[332]
		cGVHD	Reduced cGVHD score and fibrosis, macrophage accumulation, and TGF-β/Smad2 in the skin Regulation of B cell immunity		[333]
Placental MSC	lung	radiation	Decreased vascular damage, inflammation and fibrosis Decreased DNA damage	EVs enriched in miR-214-3p which targeted *ATM*/p53/p21	[86]
	kidney	UUO	Decreased αSMA and collagen I Increased infiltration of Foxp3+/IL-17+ CD4+ T cells		[144]
Menstrual blood-derived stem cells	lung	bleomycin	Reversal of alveolar cell damage and fibrosis Correction of hydroxyproline, malondialdehyde, glutathione peroxidase Suppression of ROS, mitochondrial damage, apoptosis	Targeting of *LOX1* and *NLRP3* apoptosis by EV let-7	[91]
Embryonic stem cells	heart	MI	Reduced fibrosis, increased neovascularization, cardiomyocyte survival Stimulation of survival, proliferation and cardiac commitment of CPC by EV miR-294	EVs enriched in miR290-295 cluster	[221]
			Improved cardiac function, reduced fibrosis, increased capillary density	EVs purified from human embryonic stem cell-derived CPCs	[222]
			Improved but sub-optimal heart function Fibrosis more pronounced, not lessened		[223]
	liver	TAA	Reduced fibrosis, collagen density, cellular infiltrate, necrosis, apoptosis Upregulation of *MMP9, MMP13, bcl2, TGF-β1, IL-10* Downregulation of *collagen 1α1, αSMA, TIMP1, bax, TNF-α, IL2*		[289]
Telocytes	heart	MI	Reduced fibrosis, collagen deposition, increased angiogenesis		[217]
Induced pluripotent stem cells (iPSC)	heart	MI	Improved cardiac function, reduced fibrosis and apoptosis Increased expression of pro-survival *bcl-x* and *bcl-2* Stimulation of mTOR signaling	EVs from iPSC-derived cardiomyocytes	[219]
			Improved cardiac function, increased cardiomyocyte proliferation, and reduced scar size EV therapeutic effects mimicked by miR-373 in vivo In fibroblasts in vitro, miR-373 downregulated TGF-β-mediated fibrotic gene expression by targeting *GDF* and *ROCK-2*	EVs from CPCs induced from iPSCs with ISX-9. EVs enriched in miR-373/-520 family members	[220]
	liver	CCL_4_, BDL	Suppression of fibrosis and expression of *αSMA, collagen 1α1,* and *TIMP-1* EV-mediated regulation of TGF-β-induced genes in cultured HSC	EVs from iPSC that were reprogrammed from primary fibroblasts EVs enriched in anti-fibrotic miRs (miR-92a-5p, -10b-5p, -302-3p, -92b-3p)	[288]

## Abbreviations

AAN: aristolochic acid nephropathy; AD-MSC, adipose-derived MSC; AEC, amnion epithelial cells; AKI, acute kidney injury; ALD, alcoholic liver disease; AKT, protein kinase B; ALT, alanine transaminase; Ang II, angiotensin II; ARDS, acute respiratory distress syndrome; ARF, ADP-ribosylation factor; αSMA, alpha smooth muscle cell actin; ATM, ataxia telangiectasia mutated; BALF, bronchoalveolar lavage fluid; BDL, bile duct ligation; BPD, bronchopulmonary dysplasia; BM-MSC, bone marrow MSC; BMP, bone morphogenic protein; CCl_4_, carbon tetrachloride; CCN2, cell communication network factor 2/connective tissue growth factor; CDC, cardiosphere-derived cells; cGVHD, chronic graft versus host disease; CHB, chronic hepatitis B; CHC, chronic hepatitis C; CKD, chronic kidney disease; CMSC, cardiac MSC; COPD, chronic obstructive pulmonary disease; CPC, cardiac-resident mesenchymal progenitor cells; CYP2E1, cytochrome P450 2E1; DN, diabetic nephropathy; DOCA, deoxycorticosterone acetate; DR5, death receptor 5; ECFC, endothelial colony-forming cell; ECM, extracellular matrix; EMT, epithelial-to-mesenchymal transition; ERK, extracellular signal-regulated kinase; ESC, embryonic stem cell; ESCRT, endosomal sorting complex required for transport; EV, extracellular vesicle; FN, fibronectin; FZD6, Frizzled Class Receptor 6; GDF-11, growth differentiation factor-1; GEC, glomerular endothelial cell; GLUT1, glucose transporter 1; GMC, glomerular mesangial cell; GTPase, guanosine triphosphate hydrolase; HBV, hepatitis B; HCV, hepatitis C; Hh, hedgehog; HIF, hypoxia inducible factor; HNF-4α, hepatocyte nuclear factor-4α; HSC, hepatic stellate cell; HSP, heat shock protein; HTF-C, trans-fatty acids, fructose, and cholesterol; huR, human antigen R; iCM, iPSC-derived cardiomyocytes; IGF-1, insulin-like growth factor; IGF-1R, IGF-1 receptor; IL, interleukin; ILV, intraluminal vesicle; iNOS, inducible nitric oxide synthase; IPF, idiopathic pulmonary fibrosis; iPSC, induced pluripotent stem cells; I/R, ischemia/reperfusion; KC, Kupffer cell; lnc, long noncoding; LOX-1, lectin-like oxidized low-density lipoprotein scavenger receptor-1; LPS, lipopolysaccharide; LSEC, luminal sinusoidal endothelial cell; MALAT, metastasis-associated lung adenocarcinoma transcript; MAPK, mitogen-activated protein kinase; MenSC, menstrual blood-derived stems cells; MFG-E8, milk fat globule-epidermal growth factor-factor 8; MI, myocardial infarction; miR, microRNA; MMP, matrix metalloprotease; MSC, mesenchymal stem cell; mTOR, mechanistic target of rapamycin; MVB, multivesicular body; NAFLD, non-alcoholic fatty liver disease; NASH, non-alcoholic steatohepatitis; NF-κB, nuclear factor kappa-light-chain-enhancer of activated B cells; NLRP3, NLR family pyrin domain containing 3; Nrf, nuclear factor erythroid 2-related factor; nSMase2, neutral sphingomyelinase 2; PDAC, pancreatic ductal adenocarcinoma; PDGF, platelet-derived growth factor; PDGFR, PDGF receptor; PD-L1, programmed death-ligand 1; PKM2, pyruvate kinase M2; PMN, polymorphonuclear; PMSC, placental MSC; PPAR, peroxisome proliferator-activated receptor; PSC, pancreatic stellate cell; PTEN, phosphatase and tensin homolog; Rictor, rapamycin-insensitive companion of mTOR; RISC, RNA-induced silencing complex; ROCK, rho-associated protein kinase; ROS, reactive oxygen species; S1P, sphingosine 1-phosphate; Shh, sonic hedgehog; SHP2, Src homology region 2 domain-containing phosphatase-2; SIRT, sirtuin; SK1, sphingosine kinase 1; SM, systemic mastocytosis; Smad, small mothers against decapentaplegic; SOCS, suppressor of cytokine signaling; SOD1, superoxide dismutase 1; Sparc, secreted protein acidic and rich in cysteine; SSc, systemic sclerosis; STAT, signal transducer and activator of transcription; stz, streptozotocin; TAA, thioacetamide; TEC, tubular epithelial cells; TGF-β, transforming growth factor beta; TGF-βR1, Type 1 TGF-β receptor; TIMP, tissue inhibitor of MMP; TLR, Toll-like receptor; TNF-α, tumor necrosis factor-α; TSG101, tumor susceptibility gene 101; UMSC, umbilical cord MSC; UTR, untranslated region UUO, unilateral ureteral obstruction; VEGF, vascular endothelial growth factor; WJ-MSC, Wharton’s jelly MSC; Wnt, wingless/integrated; YAP, yes associated protein.
